# Bacteriophage vB_SalS_KY05 controls *Salmonella* in poultry without disrupting cecal microbiota composition

**DOI:** 10.1080/01652176.2026.2617464

**Published:** 2026-01-21

**Authors:** Wun-Yuan Wu, Reuben Wang, Yu-Ning An, Yuan-Yu Lin

**Affiliations:** a Department of Animal Science and Technology, National Taiwan University, Taipei City, Taiwan; b Institute of Food Safety and Health, College of Public Health, National Taiwan University, Taipei, Taiwan; c Mater of Public Health Program (MPH), College of Public Health, National Taiwan University, Taipei City, Taiwan; d (GIP-TRIAD) Global Innovation Joint-Degree Program, International Joint Degree Master’s Program in Agro-Biomedical Science in Food and Health, College of Medicine, National Taiwan University, Taipei, Taiwan

**Keywords:** *Salmonella*, bacteriophage, antibiotic alternative, poultry

## Abstract

Salmonellosis is a major food safety challenge in poultry production, requiring effective antibiotic alternatives. We isolated a polyvalent *Tequintavirus*, vB_SalS_KY05, from a Taiwanese poultry farm and evaluated its biological properties and *in vivo* efficacy against *Salmonella enterica* serovars Typhimurium and Enteritidis, and *Escherichia coli*. The objective of this study was to comprehensively characterize vB_SalS_KY05 and assess its potential as a biocontrol agent for poultry farming. Genome analysis confirmed a lytic lifestyle with no detectable virulence or antimicrobial resistance genes. The phage remained stable at pH 4–10, at 41 °C, and in water for 7 days. For improved biosafety, large-scale amplification was achieved by switching the propagation host to non-pathogenic *E. coli* K12. *In vivo*, SPF chickens challenged with *S.* Typhimurium received low-dose (10^5^ PFU/mL) or high-dose (10^8^ PFU/mL) phage *via* drinking water. Low-dose treatment reduced splenic *Salmonella*, improved albumin-to-globulin ratios, and enriched beneficial taxa like *Lactobacillus crispatus* and *Blautia coccoides*. In contrast, high-dose treatment resulted in phage–bacteria coexistence and increased potentially harmful taxa including *Erysipelatoclostridium*. Overall, cecal microbiota composition remained largely unchanged. These findings indicate vB_SalS_KY05 is a promising biocontrol candidate, highlighting the importance of dose optimization to enhance pathogen control while preserving gut microbiota stability in poultry production.

## Introduction

Salmonellosis is a globally important zoonotic foodborne disease, and poultry products such as eggs and meat are key vehicles of human infection. In Taiwan, *Salmonella* accounts for approximately 30.7% of acute diarrhea cases caused by bacterial pathogens, ranking second only to *Staphylococcus aureus* (Liao et al. 2024). Among warm-blooded animals and humans, most infections are caused by *Salmonella enterica* subsp. *enterica*, with serovars Enteritidis and Typhimurium being the most prevalent and clinically relevant (Maciel et al. 2017; Shaji et al. 2023). Although national surveillance data for Taiwanese poultry farms are dated, the most recent accessible survey reports a distinct serovar distribution: *S.* Albany (21.4%) was most prevalent, followed by *S.* Typhimurium (19.4%), *S.* Livingstone (8.3%), *S.* Schwarzengrund (8.0%), and *S.* Enteritidis (5.8%) (Lin 2018). This pattern differs from that in many Western countries, where *S.* Enteritidis, *S.* Infantis, and *S.* Typhimurium typically predominate (EFSA and ECDC 2025), but still highlights *S.* Typhimurium and *S.* Enteritidis as epidemiologically relevant serovars in Taiwan. Poultry not only act as reservoirs for *Salmonella* but also facilitate its spread through both horizontal and vertical transmission, leading to contamination in hatcheries, hatching eggs, and end-consumer products (Nair and Kollanoor Johny 2019; Ijaz et al. 2021; Shaji et al. 2023). This contamination poses a substantial challenge to achieving high-quality poultry products, especially those intended for raw consumption, such as raw-grade eggs.

The widespread use of antibiotics in animal husbandry has contributed to the rise of multidrug-resistant (MDR) bacteria, posing serious public health risks (Kirbis and Krizman 2015; Wang et al. 2020). As antibiotic-free farming becomes a global trend, safe and effective alternatives, such as bacteriophages, are urgently needed to maintain disease control. Bacteriophages, which are viruses that specifically infect bacteria, are emerging as promising biocontrol agents for managing *Salmonella* in poultry. Compared to broad-spectrum antibiotics, bacteriophages offer advantages including host specificity, self-replication at the infection site, and minimal disruption to the animal host’s microbiota (Loc-Carrillo and Abedon 2011; Lin et al. 2017). Native phages—those isolated from local environments—may be particularly effective, as they are adapted to infect endemic bacterial strains under real-world conditions (Dafale et al. 2015).

Countries such as Korea, China, and members of the European Union are actively advancing bacteriophage research, spanning from isolation to field application, while simultaneously developing regulatory frameworks to support bacteriophage-based solutions (Yang et al. 2023; Zia and Alkheraije 2023). A policy report from the European Commission emphasized the importance of establishing bacteriophage databases in developing countries to facilitate the future development of bacteriophage-based biocontrol products and regulatory standards (European Commission, Joint Research Centre 2024). Bacteriophages can be applied both pre-harvest, such as in feed additives and sanitation of farming facilities, and post-harvest, including on ready-to-eat meat products. In the United States, the Food and Drug Administration has recognized several commercial bacteriophage products as GRAS (Generally Recognized as Safe), including ListShield, SalmoFresh, and PhageGuard. More recently, the European Commission approved BAFASAL, developed by Proteon Pharmaceuticals, as the first bacteriophage-based zootechnical feed additive for poultry (European Commission 2025).

Bacteriophage-based interventions against *Salmonella* have been explored as feed additives (Clavijo et al. 2019; Wójcik et al. 2020; Lee et al. 2021; Sarrami et al. 2023) and environmental sanitizers (Sevilla-Navarro et al. 2020; Evran et al. 2022; Korzeniowski et al. 2022) in poultry production, yet *in vivo* outcomes are often complex because of host–phage–microbiota interactions. Unlike conventional antibiotics, therapeutic phages are self-replicating agents that follow ‘single-hit’ kinetics and can remain effective at relatively low initial doses (Loc-Carrillo and Abedon 2011). *In vitro*, *Salmonella* phages have shown measurable efficacy across a wide range of input titers (10^3^–10^12^ PFU/mL) with responses that are not strictly dose dependent (Sevilla-Navarro et al. 2020), and intestinal phage–host studies highlight spatial heterogeneity and persistent phage–bacteria coexistence, making *in vivo* dynamics difficult to predict (Chae 2023). On this basis, we deliberately compared a low and a high vB_SalS_KY05 dose (10^5^ vs.10^8^ PFU/mL in drinking water), spanning three orders of magnitude relative to the *Salmonella* challenge dose, to investigate how widely separated input titers influence therapeutic efficacy and phage–bacteria interactions in chickens. The lower dose approximates a practically achievable field dose, whereas the higher dose represents a near-saturating input commonly used in experimental phage therapy.

Results of poultry phage studies have varied depending on the bacterial challenge model, administration route, and dosage. Critical questions remain as to whether phages, including vB_SalS_KY05, can stably persist in the poultry gastrointestinal tract, maintain infectivity against *Salmonella in vivo*, and modulate inflammatory responses in the host. In addition, few studies explicitly connect *in vitro* phage characteristics, such as physicochemical stability, genomic features, and host inhibition profiles, with their *in vivo* efficacy in poultry models. This study aims to address these gaps by comprehensively evaluating the efficacy and biological impact of vB_SalS_KY05 in controlling *S.* Typhimurium in experimentally infected chickens.

Concerns have also been raised regarding the production of therapeutic phages on pathogenic hosts because of potential contamination with bacterial components (Torres-Acosta et al. 2019). In this study, we exploited the polyvalent nature of vB_SalS_KY05 to switch its production host to a non-pathogenic *Escherichia coli* strain, thereby improving biosafety by avoiding amplification on the pathogenic host and reducing the risk of contamination during large-scale preparation. The phage was administered *via* drinking water, a practical and scalable route for poultry application, and the two dosage levels described above were used to assess phage stability, intestinal colonization, effects on host immune responses, and gut microbiota composition. By integrating both mechanistic and applied perspectives, this work provides insight into the potential of locally sourced bacteriophages as safe and effective antimicrobial alternatives in poultry production systems.

## Materials and methods

### Phage isolation and purification

Fecal samples were collected from a poultry farm in Chiayi, Taiwan. Samples were mixed with tryptic soy broth (TSB; HiMedia, India) and incubated at 37 °C with shaking at 180 rpm. The mixture was centrifuged at 10,000 ×*g* for 10 min at 4 °C, and the supernatant was filter-sterilized using a 0.22-μm PES syringe filter (Sigma-Aldrich, US). Potential phage presence was screened *via* spot test: 10 μL of filtrate was spotted onto a 1.5% tryptic soy agar plate (TSA) overlaid with 0.7% soft agar with 1 mM CaCl_2_ (Hayashi Pure Chemical Ind., Ltd., Japan) seeded with *Salmonella* Typhimurium ATCC 19585 (*S.* Typhimurium). Plaques from the spot test were resuspended in TSB and vortexed vigorously. Serial dilutions were prepared, and phages were purified using the double agar overlay assay (DLA). For DLA, 100 μL of phage lysate was combined with 400 μL of an overnight (16–18 h) *S.* Typhimurium, incubated statically for 15 min to allow phage adsorption, mixed with soft agar, poured onto TSA, and incubated at 37 °C overnight. Purified phages were propagated by adsorption on TSA with TSB for 4 h. Lysates were pooled, centrifuged at 5,000 × *g* for 10 min at 4 °C, filtered through a 0.22-μm filter, and stored at 4 °C.

### Titer determination and propagation

Phage titers were determined using an overnight *S.* Typhimurium culture as the host. Initial titers were estimated by serial dilution and spot test, followed by precise quantification *via* DLA. For experimental use, phages were prepared with *S.* Typhimurium as the host. These were propagated *via* DLA, adsorbed, and filter-sterilized as described, consistently yielding 10^10^ PFU/mL lysates. All the titering experiments were performed in triplicate.

### Morphological observation by transmission electron microscopy

High-titer phage lysate (10^10^ PFU/mL) was precipitated with 2.5 M NaCl and 20% PEG-8000 (Sigma-Aldrich, U.S.) (1:4, v/v) overnight at 4 °C, followed by centrifugation at 10,000 × g for 30 min at 4 °C. The pellet was washed with SM buffer (10 mM MgSO_4_, 100 mM NaCl, 0.01% w/v gelatin, 50 mM Tris-HCl, pH 7.5). The phage suspension was dropped onto 200-mesh carbon-coated copper grids (EMR Integrated Solutions, Ireland) for 60 s, excess liquid was removed with filter paper, and the grids were then negatively stained with 2% uranyl acetate for 5 s (EMS, U.S.). Phage morphology was observed using a Tecnai T12 transmission electron microscope (TEM; FEI, U.S.). Structural dimensions were measured with ImageJ software (NIH, U.S.).

### Host range determination

Host range was assessed *via* spot test using *Salmonella* Typhimurium ATCC (American Type Culture Collection, U.S.) 19585, 12947, and 23566; *S.* Enteritidis ATCC 13076; other serovars including *S.* Postadam ATCC 25957 and *S. diarizonae* ATCC 12325; field isolated strains *S.* Albany, *S.* Derby, *S.* Livingstone, *S.* Newport, and *S.* Schwarzengrund (provided by Dr. Chung-Hsi Chou, National Taiwan University); and *Escherichia coli* ATCC 11303 (strain B), *E. coli* K12 BCRC (Bioresource Collection and Research Center, Taiwan) 51445, *Escherichia coli* ATCC 43895, *Escherichia coli* ATCC 23546, and *Staphylococcus aureus* BCRC 13077. Efficiency of plating (EOP) was calculated using the DLA with the formula: EOP = titer on target strain/titer on the host strain (*S.* Typhimurium ATCC 19585).

### Whole-genome sequencing of vB_SalS_KY05

High-titer phage lysate was precipitated with 2.5 M NaCl and 20% PEG-8000 (Sigma-Aldrich, U.S.) (1:4, v/v) overnight at 4 °C, followed by centrifugation at 10,000 × g for 30 min at 4 °C. The pellet was resuspended in 500 µL 5 mM MgSO_4_ and treated with 2.5 µL DNase I and RNase A (Thermo Scientific, U.S.) at 37 °C for 1 h. Protein digestion was performed using proteinase K, SDS, and EDTA at 60 °C for 1 h. Genomic DNA was extracted using phenol: chloroform: isoamyl alcohol (25:24:1), precipitated with ethanol and sodium acetate, and resuspended in ddH_2_O. DNA quality was verified with a NanoDrop spectrophotometer. Whole-genome sequencing was conducted using the ONT PromethION 24 platform with long-read library preparation *via* the KAPA HyperPlus Kit (KAPA Biosystems, Roche). Basecalling was performed using Dorado with a quality filter of *Q* > 10. *De novo* assembly was carried out using Canu (Koren et al. 2017), followed by polishing and quality assessment with QUAST (Gurevich et al. 2013) and BUSCO (Seppey et al. 2019). Gene prediction and annotation were performed with Prokka (Seemann 2014) and DIAMOND (v0.9.22) (Buchfink et al. 2015) against the NCBI NR database (E-value ≤ 1E − 5). Additional annotation using PhageScope (Wang et al. 2024) identified putative lifestyle, virulence, and resistance genes from Virulence Factor Database (VFDB) and Comprehensive Antibiotic Resistance Database (CARD). Genome visualization was performed with SnapGene, and the resulting genome map is provided in the Supplementary Data.

### Bacterial growth inhibition in liquid medium

About 100 μL of phage lysate was serially diluted and mixed with 100 μL of freshly cultured target bacteria (OD_600_ = 0.1, ∼10^7^ CFU/mL) at MOI (multiplicity of infection) of 0.1, 1, and 10. Mixtures were incubated with shaking at 37 °C in a microplate reader (BioTek Instruments, U.S.), and OD_600_ was measured every 15 min for 12 h.

### Stability assessment

The stability assay was performed according to Governal and Gerba (1997), Ravindran (2013) and Lorenzo-Rebenaque (2021), with modifications. For pH stability, phage lysate (200 μL) was added to 800 μL of pH-adjusted TSB (pH 2–10) and incubated at 37 °C for 1 h. For thermal stability, phage lysate (200 μL) was mixed with 800 μL TSB and incubated at room temperature (22 °C), representing housing conditions, simulated chicken gut temperature (41 °C) (Prinzinger et al. 1991), or a potential heat-inactivation temperature (65 °C) for 30 or 60 min. For water stability, phage lysate (100 μL) was diluted in 990 μL of sterile-filtered (0.22 μm) reverse osmosis (RO) water, double-distilled water (ddH_2_O), or tap water and stored at room temperature (22 °C) for 7, 14, 21, 28 and 90 days. Bacteriophage titers were determined at each time point.

### Host switching evaluation

Phage production was tested in liquid culture: 100 μL lysate was mixed with 1 mL 10 mM CaCl_2_ and 4 mL of *S.* Typhimurium and *E. coli* K12, incubated at 37 °C with shaking at 140 rpm overnight, and titers were determined as described. Lysates in the recipe were tested at low (10^6^ PFU/mL) and high (10^8^ PFU/mL) titers. Bacterial cultures were prepared in log-phase (2.0 mL overnight broth in 18.0 mL TSB, incubated for 2 h, adjusted to OD_600_ = 0.2) or overnight.

### Applying *Salmonella*-infected chicken with vB_SalS_KY05 *via* drinking water

**Figure 1.**
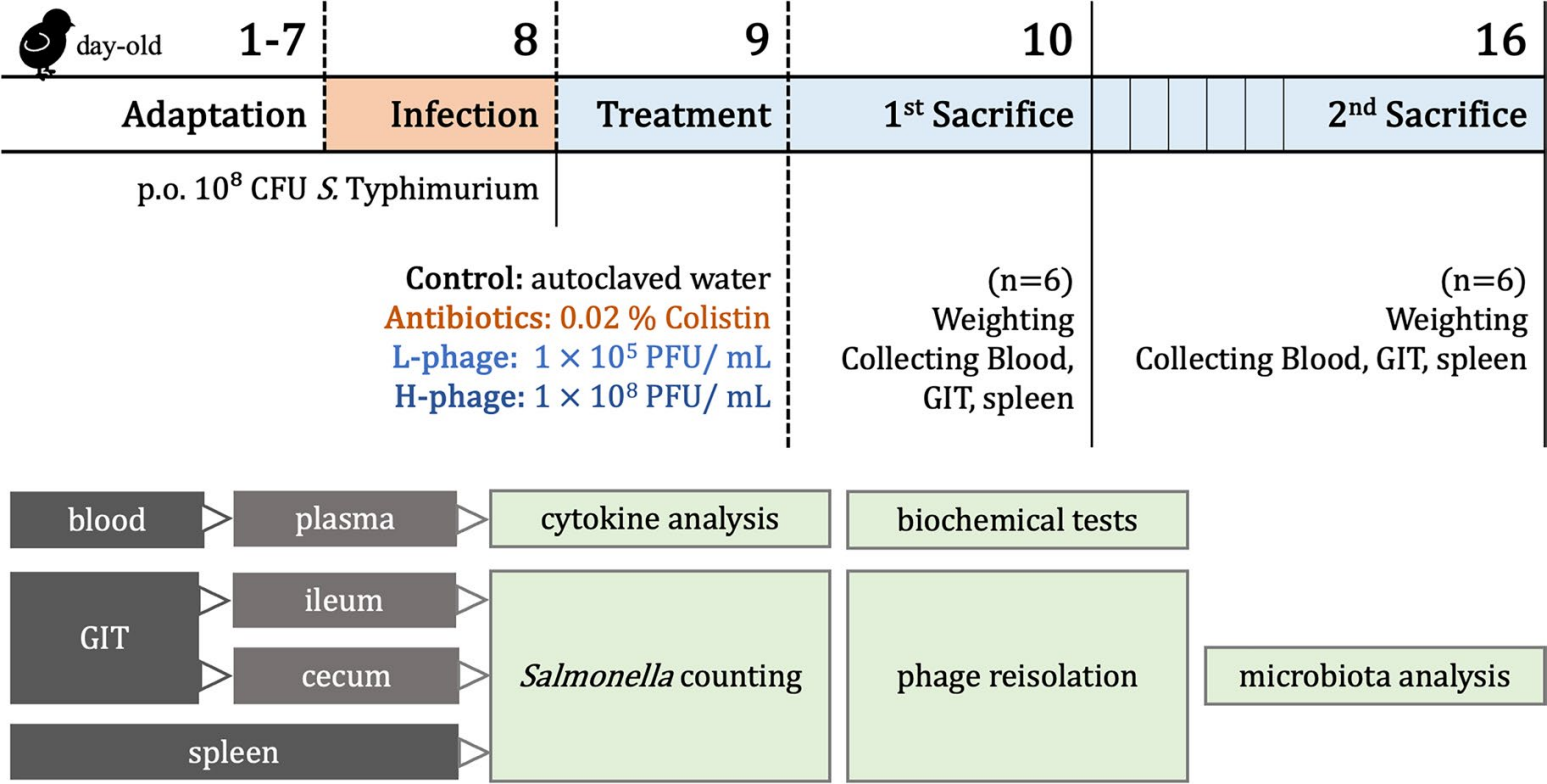
Experimental design for administration of vB_SalS_KY05 *via* drinking water to *Salmonella*-infected chickens. After a 7-day adaptation period, day-old chicks were orally challenged with 10^8^ CFU of *Salmonella* Typhimurium ATCC 19585 and then allocated to four treatment groups receiving different drinking-water formulations: Control, autoclaved water; Colistin, 0.02% Colistin; L-phage, 1 × 10^5^ PFU/mL vB_SalS_KY05; H-phage, 1 × 10^8^ PFU/mL vB_SalS_KY05. After 1 day (d-10) and 7 days (d-16) of treatment, six chicks per group were euthanized for sampling, as outlined in the schematic.

The experimental design is illustrated in Figure 1. A total of 48 one-day-old specific pathogen-free (SPF) Babcock Leghorn chicks were used in this experiment. The chicks were obtained from JD-SPF Biotech (Taiwan) and housed in the ABSL-2 poultry facility of the Animal Resource Center at National Taiwan University. Chickens were provided ad libitum access to water and a drug-free commercial chick starter diet (Taiwan Sugar Corporation, Taiwan). The average ambient temperature in the facility was 21.36 ± 0.45 °C, with an average relative humidity of 56.81 ± 6.84%. Each pen was equipped with a brooding lamp for supplemental heat. All animal procedures were approved by the Institutional Animal Care and Use Committee of National Taiwan University (Approval No. NTU-IACUC-11300072).

Chicks were randomly allocated into four groups based on body weight, with an average initial weight of 38.07 ± 0.21 g and no significant difference between groups (*p* = 0.97). After a 7-day acclimation period, each chick was orally inoculated *via* gavage on day 8 with 1 mL of *Salmonella enterica* serovar Typhimurium ATCC 19585 at 10^8^ CFU/mL. At 24 h post-infection, chickens received drinking-water treatments according to group assignment: (1) Control group, sterile water; (2) Antibiotic group (Colistin), 0.02% Colistin-120 (China Chemical and Pharmaceutical, Taiwan); (3) Low-dose phage group (L-phage),10^5^ PFU/mL of vB_SalS_KY05; and (4) High-dose phage group (H-phage), 10^8^ PFU/mL of vB_SalS_KY05. For all groups, the drinking water was prepared with 1% (v/v) TSB, which served as the diluent for both the phage lysate and Colistin. The working concentration of Colistin was prepared following the manufacturer’s instructions, corresponding to a daily dose of 50 mg/kg body weight (BW/day) as recommended for poultry. Chickens were randomly picked and euthanized on days 1 and 7 post-treatment (*n* = 6). After weighing and blood collecting, chickens were euthanized by carbon dioxide asphyxiation, and samples were collected for further analysis.

### *Salmonella* load and bacteriophage re-isolation

On the day of euthanasia, fresh samples of the ileum, cecum, and spleen were weighed and homogenized with 1 mL of sterile phosphate-buffered saline (PBS) using a tissue homogenizer (QIAGEN, Germany) at a shaking frequency of 5 Hz. Homogenization was carried out for 2 min in a pre-chilled rotor, repeated twice for a total of 4 min. A 100 μL aliquot of the homogenate was mixed with 400 μL of PBS and centrifuged at 5,000 × g for 5 min at 4 °C to pellet bacteria and tissue debris. From the resulting supernatant, 300 μL was filtered through a 0.22 μm syringe filter to remove remaining bacteria, yielding a potential phage-containing filtrate. This filtrate was subjected to spot assay against *S.* Typhimurium to determine phage titer.

The pellet was resuspended by pipetting, serially diluted, and plated onto *Salmonella spp.* detection pads (MC Media Pad, JNC, Japan) to quantify classical blue-colored colonies. Bacterial and phage concentrations were calculated as: (Plating volume × dilution factor)/tissue weight, yielding colony-forming units (CFU) and plaque-forming units (PFU) per gram of tissue, respectively.

### Inflammatory cytokines and the albumin to globulin ratio in chicken plasma

Whole blood was left at room temperature for 30 min to allow sedimentation, followed by centrifugation at 2,000 × g for 10 min. The resulting plasma was collected and stored at −80 °C until analysis. One day prior to testing, samples were thawed at 4 °C. Plasma concentrations of the proinflammatory cytokines interleukin (IL)-6 and IL-1β were determined using commercial chicken-specific ELISA kits (BlueGene, China), following the manufacturer’s instructions. After color development, absorbance was measured at 450 nm, and cytokine levels were calculated using standard curves generated from known concentrations of the standards: **IL-6: y = −509.39x + 93.183 (R^2^ = 0.968); IL-1β: y = −606.47x + 145.31 (R^2^ = 0.929).** Final concentrations were expressed as pg/mL of plasma. Total protein (TP) and albumin (ALB) levels were measured using a HITACHI 7180 automated wet-chemistry analyzer (Hitachi High-Tech Corporation, Tokyo, Japan) with commercial kits 463–48791 and 410–66901 (FUJIFILM Wako Pure Chemical Corporation, Japan). Globulin (GLB) concentration was calculated as: **GLB = TP − ALB.**

### 16S rRNA amplicon sequencing and bioinformatic analyses

Total DNA was extracted from cecal contents using the QIAamp PowerFecal Pro DNA Kit (QIAGEN, Germany). DNA quality was assessed using a Qubit 4.0 Fluorometer (Thermo Scientific, U.S.). Full-length 16S rRNA gene amplification and sequencing were performed using the PacBio Sequel IIe platform. Circular Consensus Sequencing was applied to generate high-fidelity (HiFi) reads with a predicted accuracy of Phred Q30 or above. HiFi reads were denoised using DADA2 (v1.20) (Callahan et al. 2016) to identify amplicon sequence variants (ASVs). Taxonomic classification of representative sequences was conducted using the feature-classifier and classify-consensus-vsearch plugins in QIIME2 (v2022.11) (Bolyen et al. 2019) against the NCBI 16S rRNA database. Alpha diversity, including observed features, Shannon’s, and Simpson’s indices, was assessed using QIIME2 and drawn with GraphPad Prism 10 (GraphPad Software, U.S.). Beta diversity was assessed using Bray-Curtis distance matrices computed *via* the microeco R package. Group-level microbial community differences were evaluated using PERMANOVA, and dimensionality reduction was performed *via* Principal Coordinates Analysis (PCoA) and Constrained Principal Coordinates Analysis (CPCoA). LEfSe analysis was used to identify significant microbial biomarkers, with an LDA score (log_10_) threshold ≥ 3.0.

### Statistical analysis

All statistical analyses were performed using GraphPad Prism 10 (GraphPad Software, USA) and Microsoft Excel v16.78 (Microsoft Corporation, USA). Normality was assessed using the Shapiro–Wilk test, and homogeneity of variance was evaluated using the F-test or the Brown–Forsythe test. For two-group comparisons, an unpaired t-test was applied when both normality and homogeneity assumptions were met (*p* > 0.05); otherwise, the Mann–Whitney U test was used. For multiple-group comparisons, one-way ANOVA followed by Tukey’s post hoc test was used if all groups met the assumptions of normality and equal variance. When these assumptions were violated, the Kruskal–Wallis test was followed by Dunn’s test. Post-hoc tests were only conducted if significant differences were detected (α = 0.05). Significant differences were indicated by asterisks or different letters. Specific statistical tests used are described in the figure legends or table footnotes.

## Results

### TEM, host range, and liquid inhibition analysis of vB_SalS_KY05

**Figure 2.**
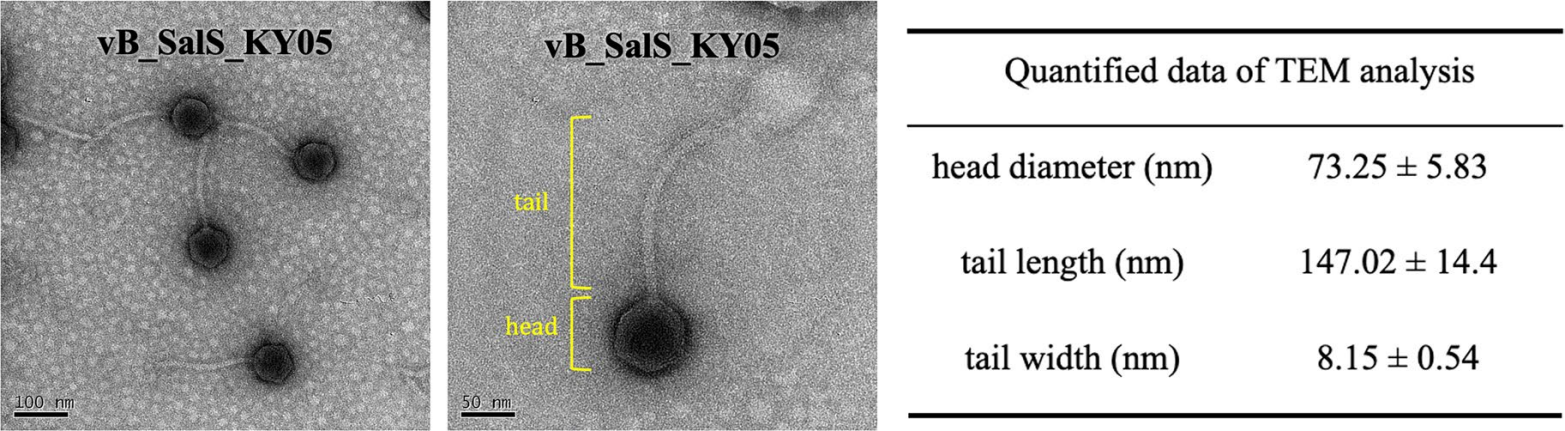
Transmission electron microscope (TEM) analysis of bacteriophages vB_SalS_KY05. TEM with a scale bar of 100 nm (left) and 50 nm (right); Quantified data of TEM analysis using Image J software, n = 3 pictures.

**Figure 3.**
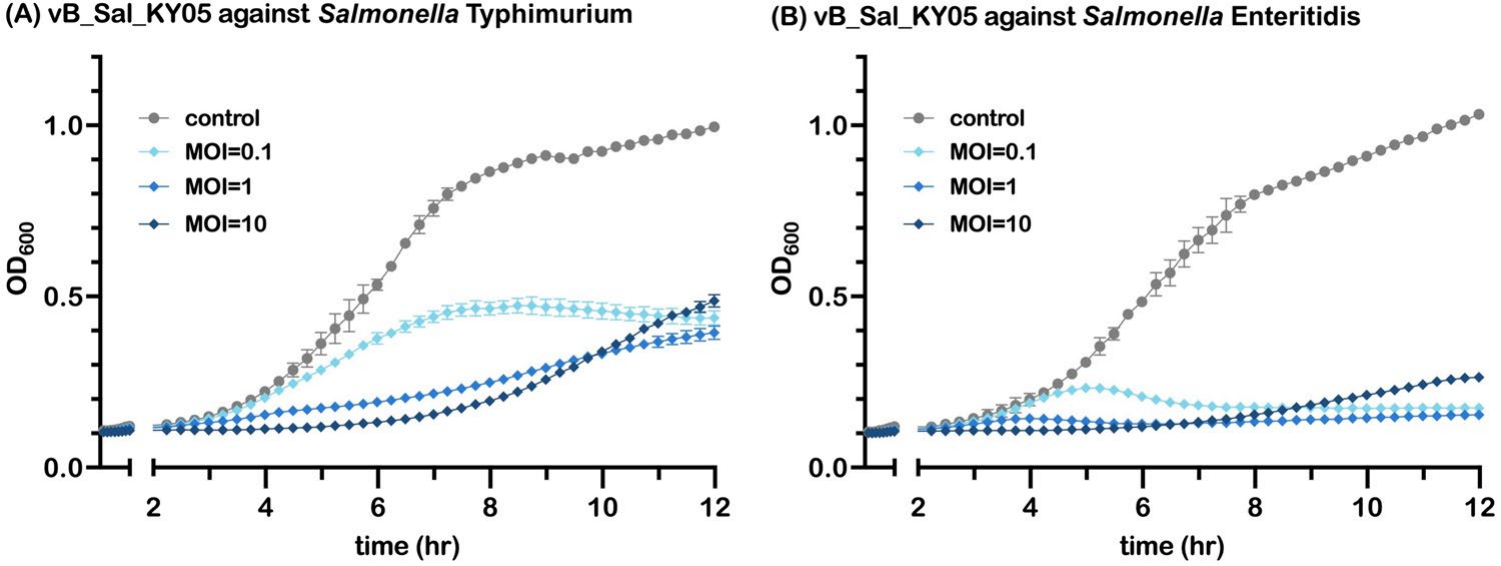
vB_SalS_KY05 Growth inhibition effect on *Salmonella* serovar Typhimurium (A) and Enteritidis (B) with MOI 0.1/1/10. Phage lysate mixed with *S.* Enteritidis (OD_600_ = 0.1, ∼10^7^ CFU/mL) at MOI (multiplicity of infection) of 0.1, 1, and 10. The optical density was measured at 15-minute intervals. Data are presented as means ± standard deviation (SD) (n = 3), and no further statistics were conducted here.

As shown in Figure 2, TEM revealed that vB_SalS_KY05 is a tailed bacteriophage with an average head diameter of 73.23 nm and a tail length of 147.02 nm. The host range indicated its polyvalent characteristic, which is capable of lysing *Salmonella* and some of *Escherichia coli*. Notably, standard strains such as *Salmonella enterica* serovar Typhimurium, Enteritidis, and Potsdam were susceptible to vB_SalS_KY05, whereas wild-type strains were not (Table 1). Herein, the EOP demonstrated comparable lytic activity against *S.* Typhimurium (1.00) and *S.* Enteritidis (0.97), with an even higher value observed for *E.* coli K12 (1.77) (data not shown).vB_SalS_KY05 also exhibited a greater ability to delay the entry of *S.* Typhimurium and *S.* Enteritidis into the logarithmic growth phase as the multiplicity of infection (MOI) increased (Figure 3). In Figure 3A, the growth curves for MOI = 10 and MOI = 1 intersected at approximately 10 h, reaching similar levels by 12 h, along with the MOI = 0.1 group. In Figure 3B, the final turbidity of *S.* Enteritidis cultures supplemented with phages showed no clear dose-dependent effect, with comparatively lower bacterial loads observed at MOI = 0.1 and MOI = 1.

**Table 1.** The host range of vB_SalS_KY05.

Strain name	Lytic degree	
*Salmonella* Typhimurium ATCC 19585	+++	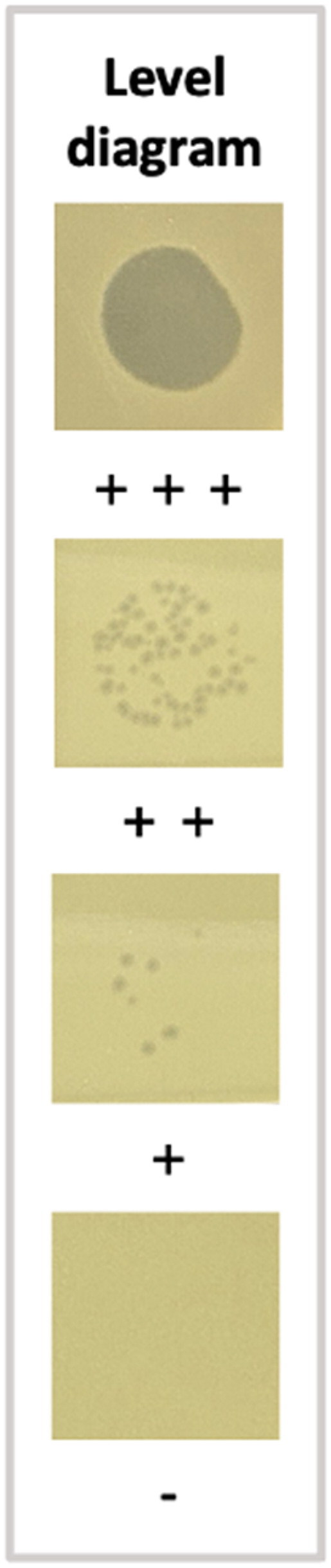
*Salmonella* Typhimurium ATCC 12947	+++	
*Salmonella* Typhimurium ATCC 23566	+++	
*Salmonella* Enteritidis ATCC 13076	+++	
*Salmonella* Potsdam ATCC 15463	+	
*Salmonella* serovar Albany (field isolates)	–	
*Salmonella* serovar Derby (field isolates)	–	
*Salmonella* serovar Livingstone (field isolates)	–	
*Salmonella* serovar Newport (field isolates)	–	
*Salmonella* serovar Schwarzengrund (field isolates)	–	
*Salmonella diarizonae* ATCC 15576	–	
*Escherichia* *coli* K12 BCRC 51445	+++	
*Escherichia coli* ATCC 11303	+++	
*Escherichia coli* ATCC 43895	–	
*Escherichia coli* ATCC 23546	–	
*Staphylococcus aureus* BCRC 13077	–	

The intensity results were recorded as follows: +++, confluent lysis; ++, semiconfluent lysis; +, individual plaque; -, no lysis. The standard strains (designated by strain numbers) were obtained from the American Type Culture Collection (ATCC, U.S.) and the Bioresource Collection and Research Center (BCRC, Taiwan). The field isolated strains, *Salmonella* serovars Albany, Derby, Livingstone, Newport, and Schwarzengrund, were provided by Dr. Chung-Hsi Chou at National Taiwan University.

### Genomic analysis of vB_SalS_KY05

vB_SalS_KY05 (GenBank accession no. PX046473) possesses a double-stranded DNA genome of 109,355 bp, with a GC content of 39% and 171 coding sequence (CDS). It is classified within the genus *Tequintavirus* (taxid: 187218), family *Caudoviricetes*. According to PhageScope analysis, vB_SalS_KY05 is a strictly lytic phage with no detected virulence factors or antimicrobial resistance genes (summarized in Table S1).

The genome can be divided into three major functional regions containing clusters of related genes. The first part (1–30,899 bp) is shorter CDS with a variety of functions, including lysis-related proteins such as holin, Rz-like spanin, and endolysin, as well as 22 tRNA genes. The second part (30,899-78,779 bp) encodes structural proteins such as capsid, tail, baseplate, and tail fiber proteins. The third part (78,779-109,335 bp) contains genes associated with host infection and genome replication. Figures are presented in the (Supplementary Material Figures S2 and S3).

### Stability evaluation of vB_SalS_KY05

**Figure 4.**
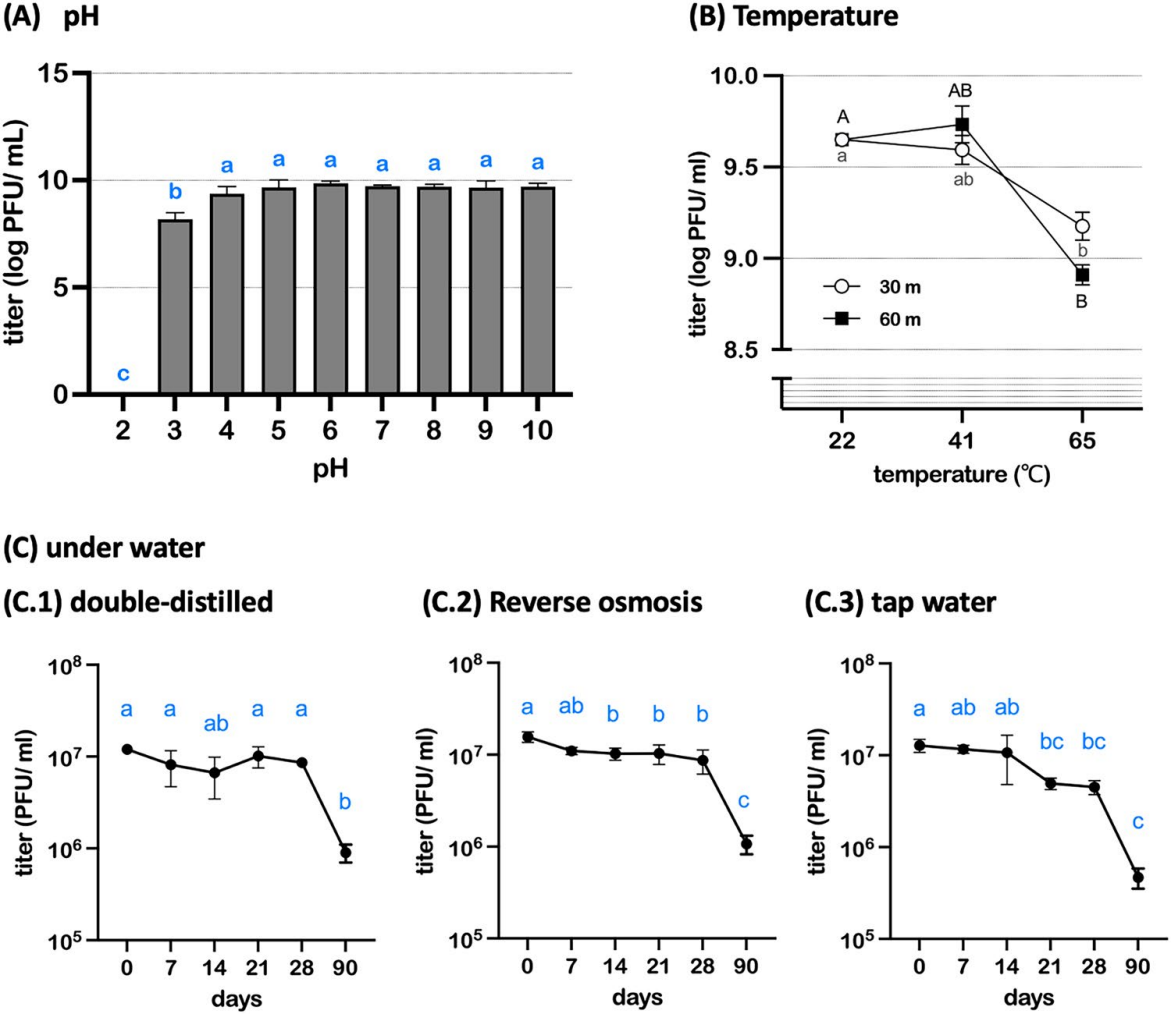
Stability of vB_SalS_KY05 at different pH values (a), temperatures (B), and aqueous environments (C). For pH stability (A), vB_SalS_KY05 suspensions were incubated in TSB adjusted to pH 2–10 for 1 h at 37 °C. Data are presented as means ± standard deviation (SD) (n = 3). Statistical comparisons were performed using one-way ANOVA followed by Tukey’s multiple-comparisons test. Different letters indicate significant differences among treatments (p < 0.05).

Figure 4 illustrates changes in the titer of vB_SalS_KY05 under varying pH, temperature, and water conditions. After 1 h of incubation at pH 3, a significant reduction in bacteriophage activity was observed, and complete inactivation occurred at pH 2. In contrast, titers remained stable across the pH range of 4–10 (Figure 4A). vB_SalS_KY05 tolerated simulated chicken core temperature (41 °C) for 1 h but underwent rapid inactivation at 65 °C within 30 min of incubation (*p* < 0.05) (Figure 4B). For water stability assessment, phage titers were maintained for at least 7 days in ddH_2_O, RO water, and tap water, with a significant decline observed by day 90 (*p* < 0.05) (Figure 4C).

### Host-switching in vB_SalS_KY05 propagation

**Figure 5.**
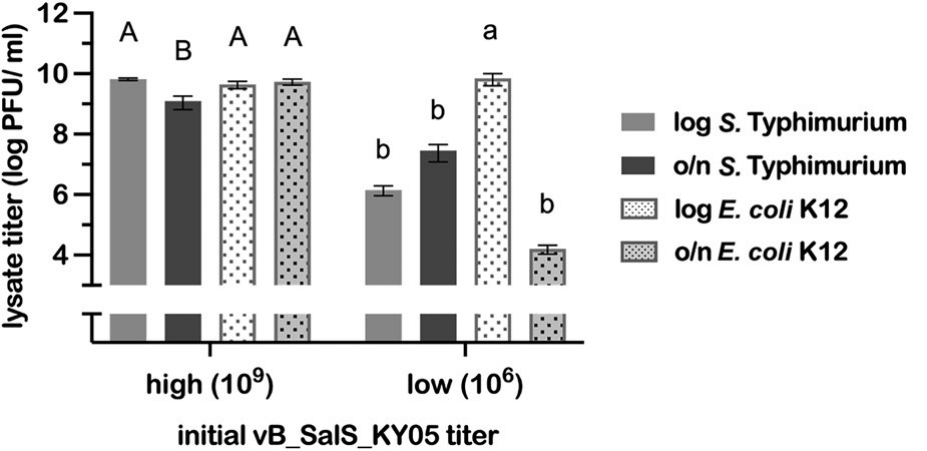
The efficiency of different bacteria serves as a host to propagate vB_SalS_KY05. Data are presented as means ± standard deviation (SD) (n = 3). Statistical comparisons were performed using the one-way ANOVA with Tukey’s multiple comparisons. Letters indicate the significant difference (*p* < 0.05) between treatments for high (10^9^ PFU/mL) or low (10^6^ PFU/mL) initial titer, and bacteria applied after overnight incubation (o/n, 16–18 hours) or at the log-phase (log, OD_600_= 0.2).

In Figure 5, bacteriophage lysates produced using different bacterial hosts, *S.* Typhimurium and *E. coli* K12, were titered on *S.* Typhimurium. When propagated at a high initial titer, vB_SalS_KY05 showed no significant difference in final titers between log-phase *S.* Typhimurium and *E. coli* K12 hosts (*p* > 0.05). In contrast, at a lower initial titer of 10^6^ PFU/mL, switching the propagation host from log-phase *S.* Typhimurium to *E. coli* K12 resulted in an approximately 1000-fold increase in phage titer (*p* < 0.05).

### Applying vB_SalS_KY05 *via* drinking water with *Salmonella*-infected chicken

#### Feed intake and body weight

**Figure 6.**
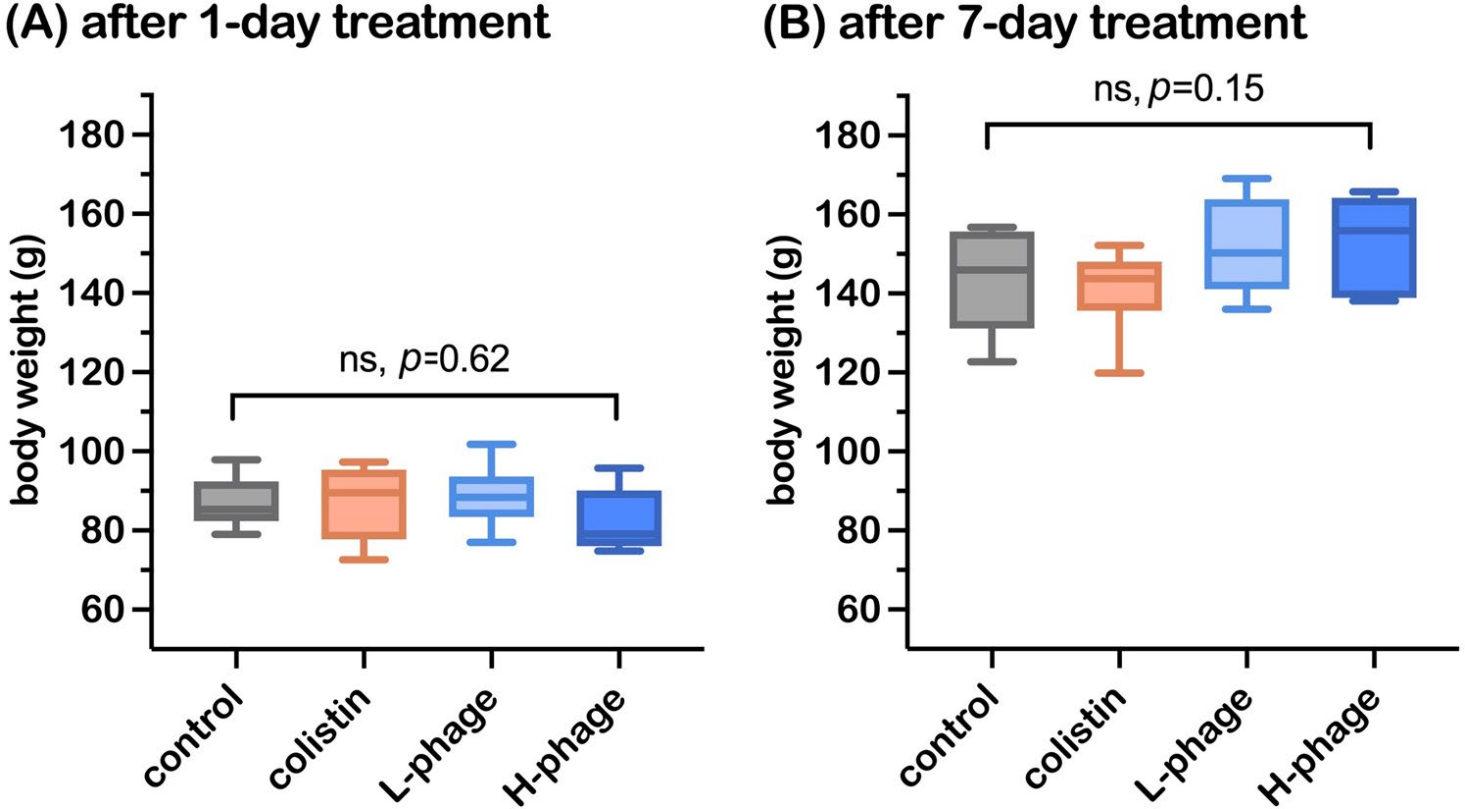
Effects of phage treatment on the body weight of SPF chicks infected with *Salmonella* Typhimurium after 1 and 7 days of treatment. Data are presented as means ± standard deviation (SD) (n = 6 per group, except for the day 1 H-phage group, where n = 5). Statistical comparisons were performed using the one-way ANOVA, and ‘ns’ indicates no significant difference. All groups were *Salmonella*-challenged. Treatments: Control, autoclaved water; Colistin, 0.02% Colistin; L-phage, 1 × 10^5^ PFU/mL; H-phage, 1 × 10^8^ PFU/mL.

During the treatment period (day 8–16), the average feed intake of the four groups was 12.11 ± 1.43 g per chick per day, with an average water consumption of 21.50 ± 1.29 mL per chick per day. At 8 days of age, all chickens were orally challenged with *S.* Typhimurium, and body weights were measured after 1 day and 7 days of treatment. As shown in Figure 6, no significant differences in body weight were detected between groups (*p* > 0.05).

#### Bacterial load and bacteriophage titer

**Figure 7.**
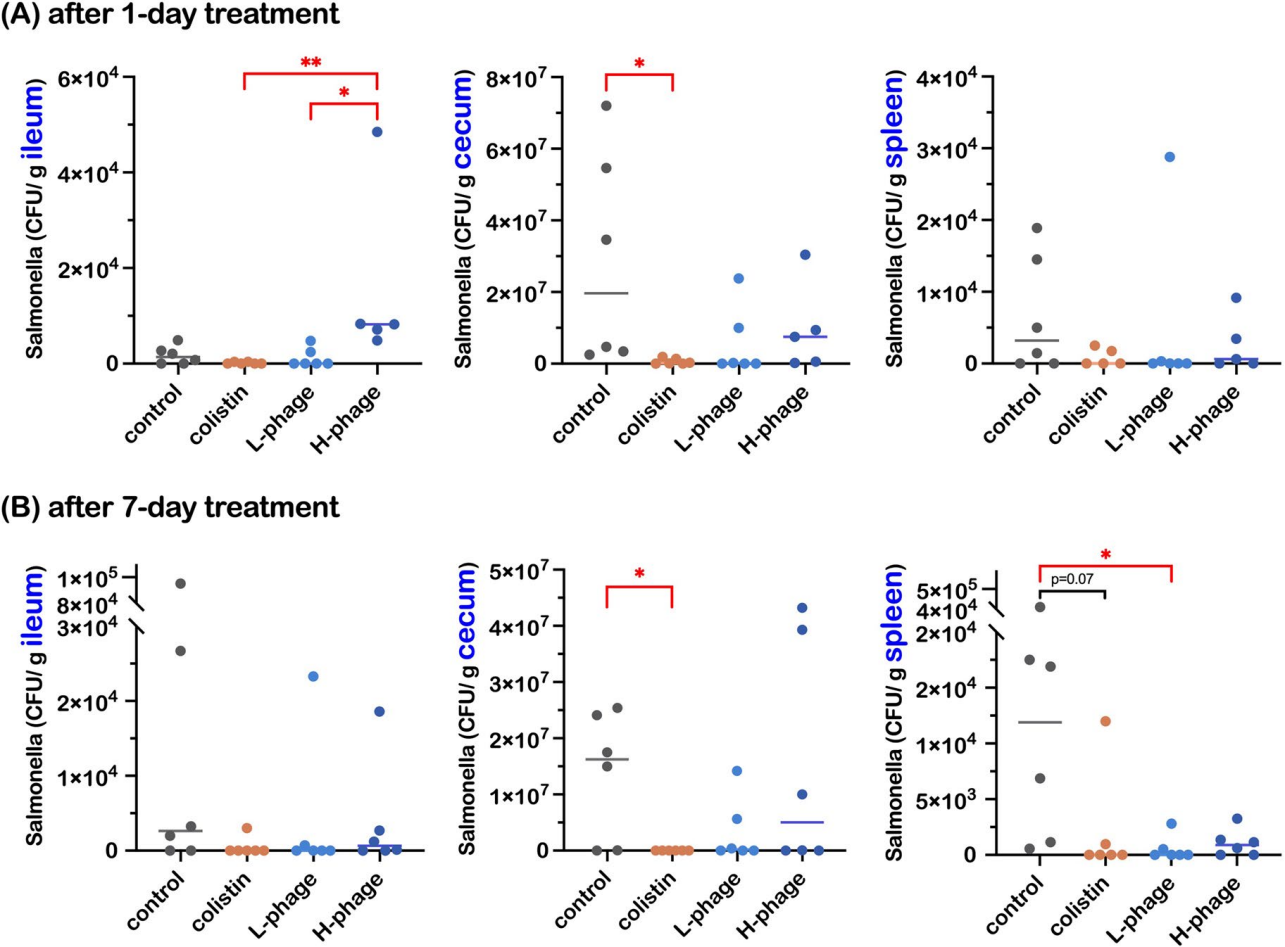
*Salmonella* Load in the ileum, cecum, and spleen of chickens after 1 and 7 days of treatment. Data are presented as medians with individual values (n = 6 per group, except for the day 1 H-phage group where n = 5). Statistical analysis was performed using the Kruskal–Wallis test followed by Dunn’s multiple comparisons test. Asterisks indicate significant differences (*p < 0.05). All groups were *Salmonella*-challenged. Treatments: Control, autoclaved water; Colistin, 0.02% Colistin; L-phage, 1 × 10^5^ PFU/mL; H-phage, 1 × 10^8^ PFU/mL.

The *Salmonella* load in the ileum, cecum, and spleen of chickens was quantified after 1 and 7 days of treatment (Figure 7). After 1 day of vB_SalS_KY05 administration, surprisingly, there were significantly higher *Salmonella* detected in the H-phage group compared to L-phage and Colistin (*p* < 0.01; *p* < 0.05). Meanwhile, Colistin, serving as a positive control, consistently showed significantly lower *Salmonella* counts in the cecum compared with the untreated control at both 1 and 7 days (*p* < 0.05). After 7 days of 10^5^ PFU/mL vB_SalS_KY05 treatment, the L-phage group displayed a significant reduction in *Salmonella* load in the spleen (*p* < 0.05), suggesting mitigation of systemic infection. However, Colistin also showed a decreasing trend in the spleen at day 7, though this did not reach statistical significance (*p* = 0.07).

**Figure 8.**
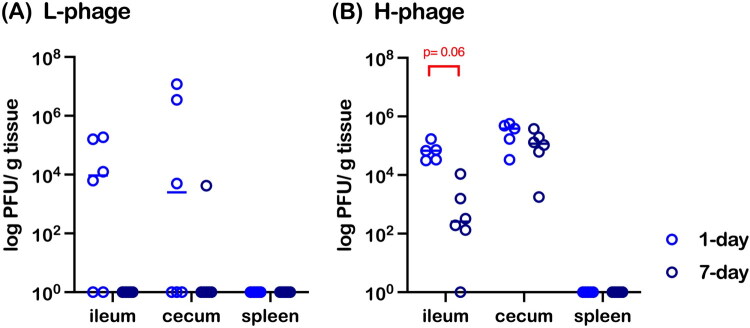
Re-Isolation of bacteriophages targeting *Salmonella* Typhimurium from the ileum, cecum, and spleen of chickens treated with low (A) or high (B) concentrations of vB_SalS_KY05. Data are presented as medians with individual values (n = 6 per group, except for the day 1 H-phage group where n = 5). Statistical analysis was performed using the Mann–Whitney U test to compare data between day 1 and day 7. Left panels: low-dose group (L-phage, 1 × 10^5^ PFU/mL); right panels: high-dose group (H-phage, 1 × 10^8^ PFU/mL).

Accordingly, the bacteriophage titer against *Salmonella* Typhimurium was assessed. In Figure 8A, only half of the chickens in the L-phage group harbored detectable phages in the ileum and cecum, with titers becoming almost undetectable by day 7. In contrast, the H-phage group (10^8^ PFU/mL) maintained a stable titer in the cecum, accompanied by a decreasing trend in the ileum (*p* = 0.06) (Figure 8B). Notably, no phages were detected in the spleen in either dosage group at either 1 or 7 days post-treatment.

#### Inflammatory cytokines and albumin-to-globulin ratio

**Figure 9.**
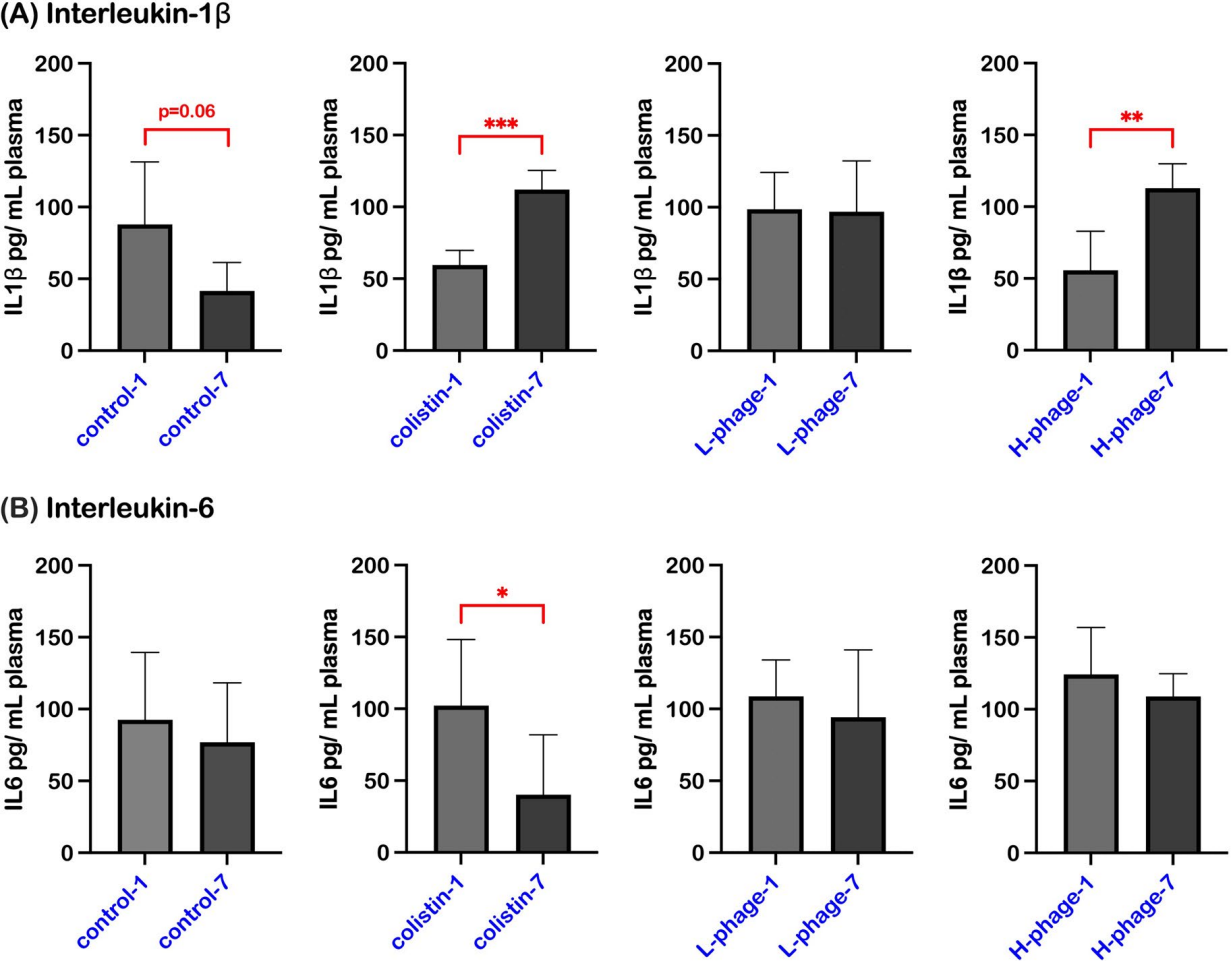
Inflammatory cytokines, (A) interleukin-1β, (B) Interleukin-6, concentrations in chicken plasma between day 1 and day 7 of treatment. Data are presented as means ± standard deviation (SD) (n = 6 per group, except for day 1 H-phage group, where n = 5). Statistical analysis was performed using an unpaired t-test. Asterisks indicate significant differences (*p < 0.05; **p < 0.01; ***p < 0.001). All groups were *Salmonella*-challenged. Treatments: Control, autoclaved water; Colistin, 0.02% Colistin; L-phage, 1 × 10^5^ PFU/mL; H-phage, 1 × 10^8^ PFU/mL.

Figure 9 shows the dynamics of inflammatory cytokines, IL-1β and IL-6, in the plasma of chickens between 1 and 7 days of treatment. In the control group, IL-1β concentration exhibited a borderline decline (*p* = 0.06); however, all treated groups, except the low-dose (L-phage) group, showed a significant increase (Colistin: *p* < 0.001; H-phage: *p* < 0.01). For IL-6, only the Colistin group exhibited a significant reduction in cytokine levels (*p* < 0.05), whereas all other groups showed no significant change between day 1 and day 7 (*p* > 0.05).

**Figure 10.**
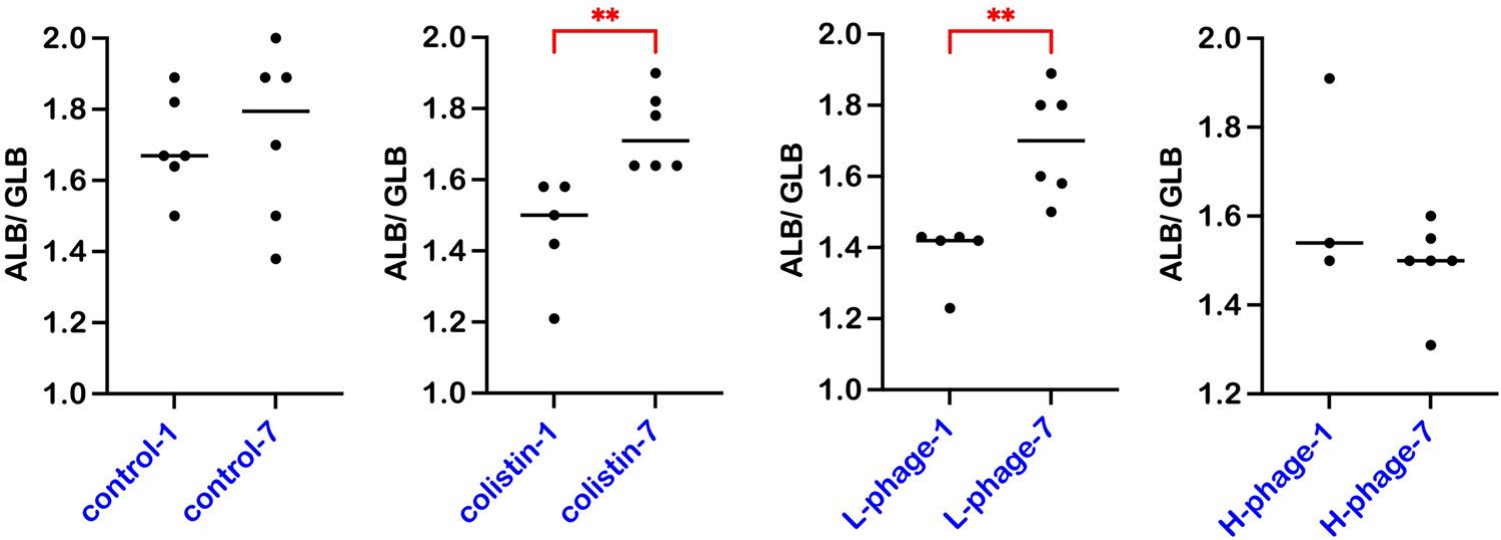
Comparison of the albumin-to-globulin ratio in chicken plasma between day 1 and day 7 of treatment. Data are presented as medians with individual values (n = 3– 6 per group). Statistical significance was assessed using the Mann–Whitney test. Asterisks indicate significant differences (*p < 0.05; **p < 0.01. Globulin levels were calculated by subtracting albumin from total protein (GLB = TP − ALB). All groups were *Salmonella*-challenged. Treatments: Control, autoclaved water; Colistin, 0.02% Colistin; L-phage, 1 × 10^5^ PFU/mL; H-phage, 1 × 10^8^ PFU/mL.

Figure 10 compares the albumin-to-globulin ratio in chicken plasma after 1 day and 7 days of treatment. Both the Colistin and L-phage groups showed a significant recovery of the ALB/GLB ratio within 7 days (*p* < 0.01). In contrast, the ratio in the control and H-phage groups remained statistically unchanged (*p* > 0.05).

### Microbiota shift

#### Diversity

**Figure 11.**
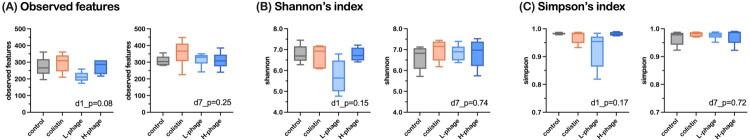
Alpha diversity indices: observed features (a), Shannon (B), Simpson (C). Treatment for 1 day (n = 5, H-phage n = 4) and 7 days (n = 6) was shown at left and right, respectively. Statistical results were analyzed by the Kruskal-Wallis test and Dunn’s multiple comparisons test. Asterisks indicate a significant difference (*p* < 0.05). Asterisks indicate significant difference (*p < 0.05). All groups were *Salmonella*-challenged. Treatments: Control, autoclaved water; Colistin, 0.02% Colistin; L-phage, 1 × 10^5^ PFU/mL; H-phage, 1 × 10^8^ PFU/mL.

Figure 11A shows the observed feature index, which represents the number of detected species without correction for sequencing depth. No significant differences were found among treatments (*p* = 0.08; *p* = 0.25). The Shannon index was used to evaluate species richness and evenness, while the Simpson index reflects the influence of dominant species through weighted values. As shown in Figures 11B and 11C, no significant differences in cecal microbiota richness were observed among treatments at either day 1 or day 7 (Shannon: *p* = 0.15; *p* = 0.74; Simpson: *p* = 0.17; *p* = 0.72).

**Figure 12.**
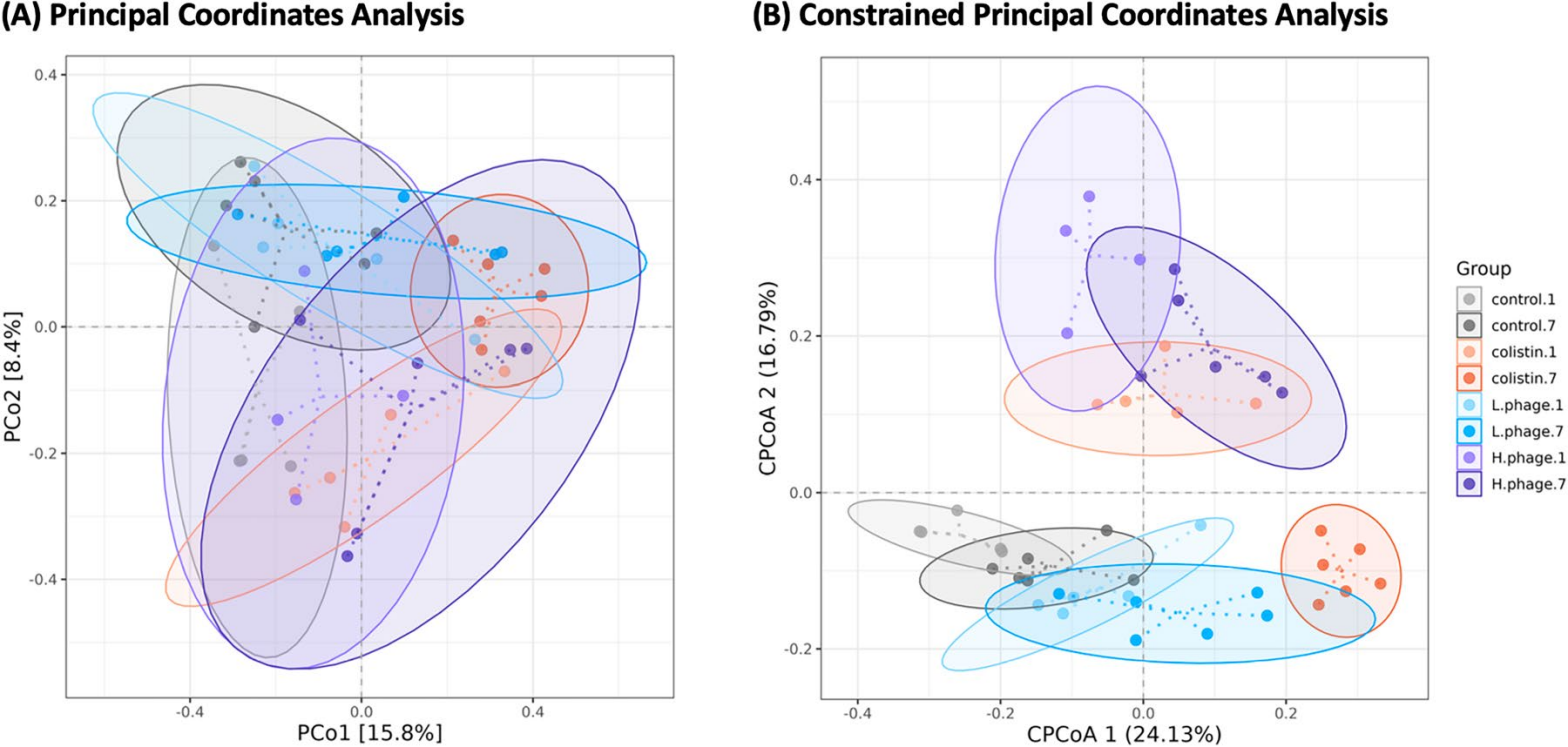
Beta diversity analysis: principal coordinates analysis (PCoA) (a), and constrained principal coordinates analysis (CPCoA) (B). Data of 1-day treatment (n = 5, H-phage n = 4) and 7-day treatment (n = 6) were used. The statistics of Bray-Curtis distance between groups showed a significant difference (PERMANOVA: p = 0.001, R^2^=0.37) as shown in PCoA. The further constrained CPCoA indicated a significant impact on treatment (p = 0.001). All groups were *Salmonella*-challenged. Control: autoclaved water for 1 (.1) or 7 (.7) days; Colistin: 0.02% Colistin for 1 (.1) or 7 (.7) days; L-phage: 1 × 10^5^ PFU/mL for 1 (.1) or 7 (.7) days; H-phage: 1 × 10^8^ PFU/mL for 1 (.1) or 7 (.7) days.

Figure 12A presents a PCoA based on Bray-Curtis distance, showing a statistically significant difference among groups according to PERMANOVA (*p* < 0.01, R^2^ = 0.37). The first principal coordinate (PCo1) explains 15.8% of the total variance, while the second (PCo2) accounts for 18.4%. Despite this, the data points form clusters with overlapping 95% confidence ellipses, indicating partial similarity among groups.

Figure 12B illustrates a constrained ordination, which reveals statistically significant group separation (*p* < 0.05). Factor 1 explains 24.13% of the variation among groups, and Factor 2 explains 16.79%, with the grouping factor accounting for 28.2% of the total variation. The 95% confidence ellipses suggest that the microbial profiles of the control and L-phage groups were more similar across timepoints, while Colistin treatment resulted in distinct differences between day 1 and day 7. Notably, samples from the H-phage group clustered within the first and second quadrants, indicating a unique microbial community structure shaped by high-dose phage treatment.

#### Top 10 relative abundance analysis

**Figure 13.**
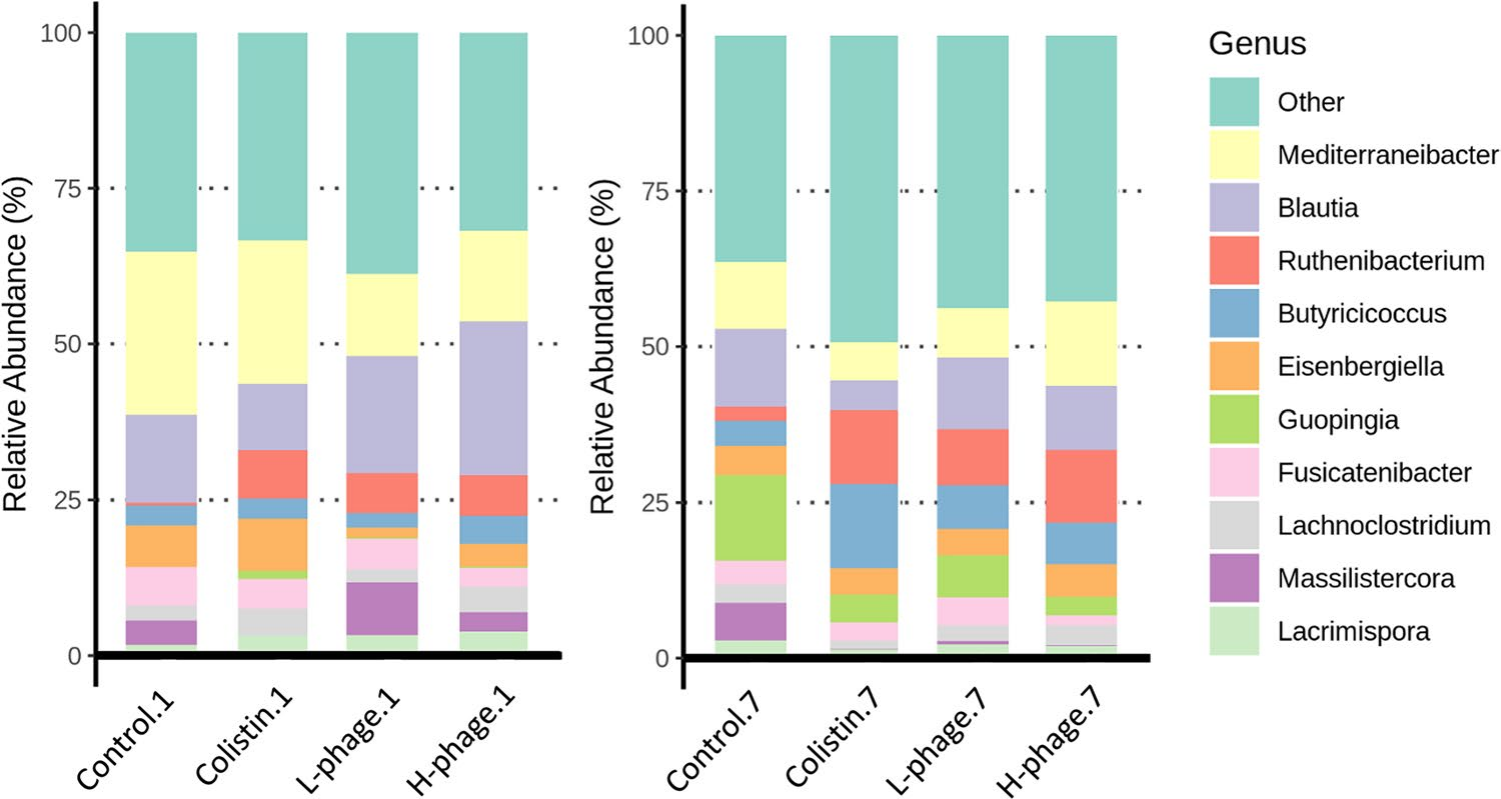
Top 10 heatmap relative abundance on the genus level. Data of 1-day treatment (n = 5, H-phage n = 4) and 7-day treatment (n = 6) were used. The left part shows the outcome after 1 day of treatment (.1), and the right part is for the 7-day treatment (.7). All groups were *Salmonella*-challenged. Control: autoclaved water for 1 (.1) or 7 (.7) days; Colistin: 0.02% Colistin for 1 (.1) or 7 (.7) days; L-phage: 1 × 10^5^ PFU/mL for 1 (.1) or 7 (.7) days; H-phage: 1 × 10^8^ PFU/mL for 1 (.1) or 7 (.7) days.

Figure 13 and Figure S1 present the top 10 dominant genera and species in each treatment group, ranked by relative abundance. In this study, over 99% of bacteria belong to Firmicutes. No significant differences were observed among groups at day 1 (*p* > 0.05). At the genus level, *Massilistercora* abundance in the control group was significantly higher than in the Colistin and H-phage groups (*p* < 0.05; *p* < 0.01), while *Butyricicoccus* abundance in the Colistin group was significantly higher than in the control group (*p* < 0.01). At the species level, the Colistin group had a significantly higher abundance of *Butyricicoccus pullicaecorum* (*p* < 0.05), and *Butyricicoccus faecihominis* abundance in the L-phage group was significantly higher than in the H-phage group (*p* < 0.05).

#### Linear discriminant analysis effect size (LEfSe)

**Figure 14.**
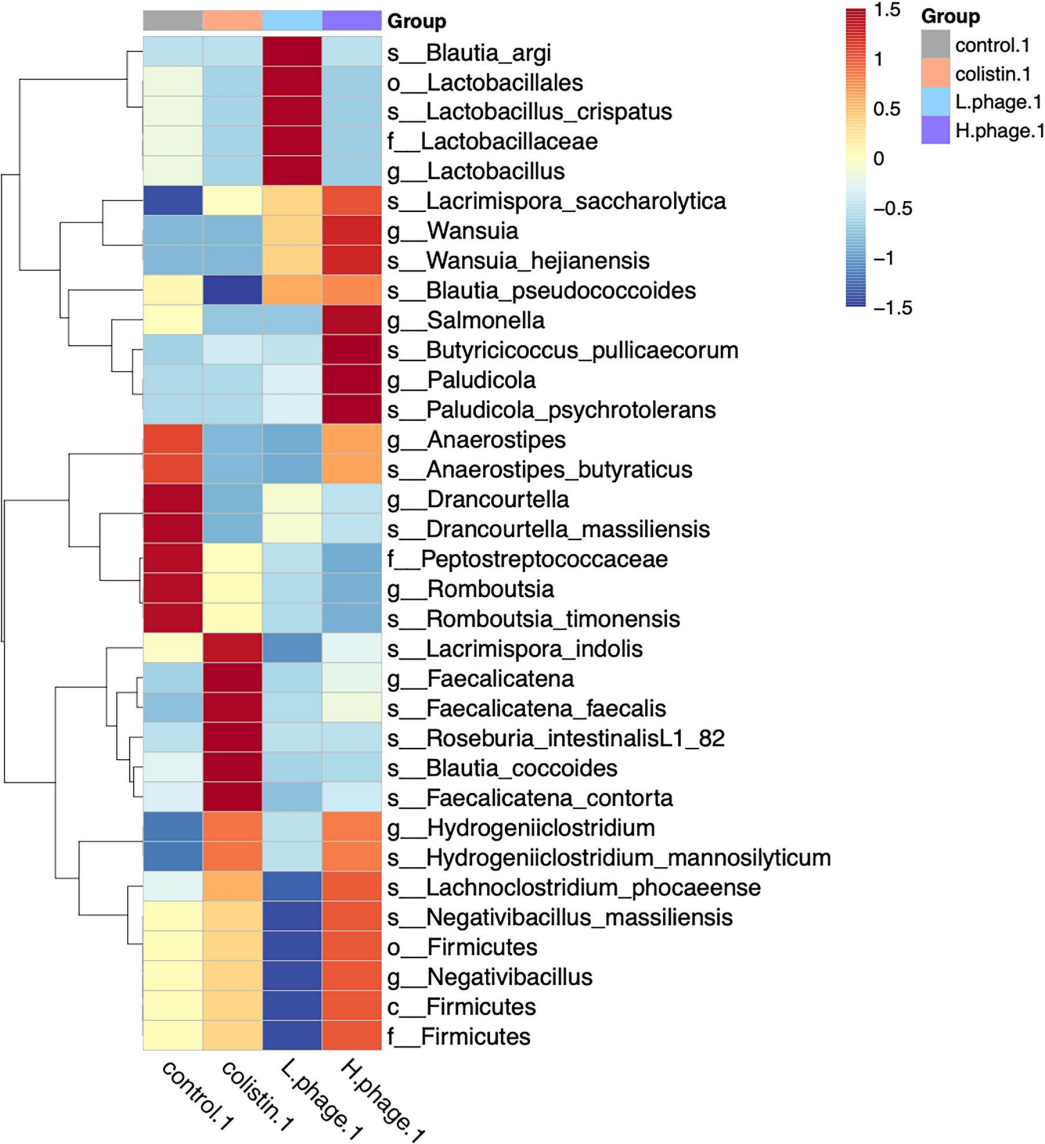
LEfSe (Linear discriminant analysis (LDA) effect size) analysis of microbiome after 1 day of treatment. Data of 1-day treatment (n = 5, H-phage n = 4) and 7-day treatment (n = 6) are used, calculated with an LDA score ≧ 3.0 were shown. All groups were *Salmonella*-challenged. Treatments: Control, autoclaved water; Colistin, 0.02% Colistin; L-phage, 1 × 10^5^ PFU/mL; H-phage, 1 × 10^8^ PFU/mL.

**Figure 15.**
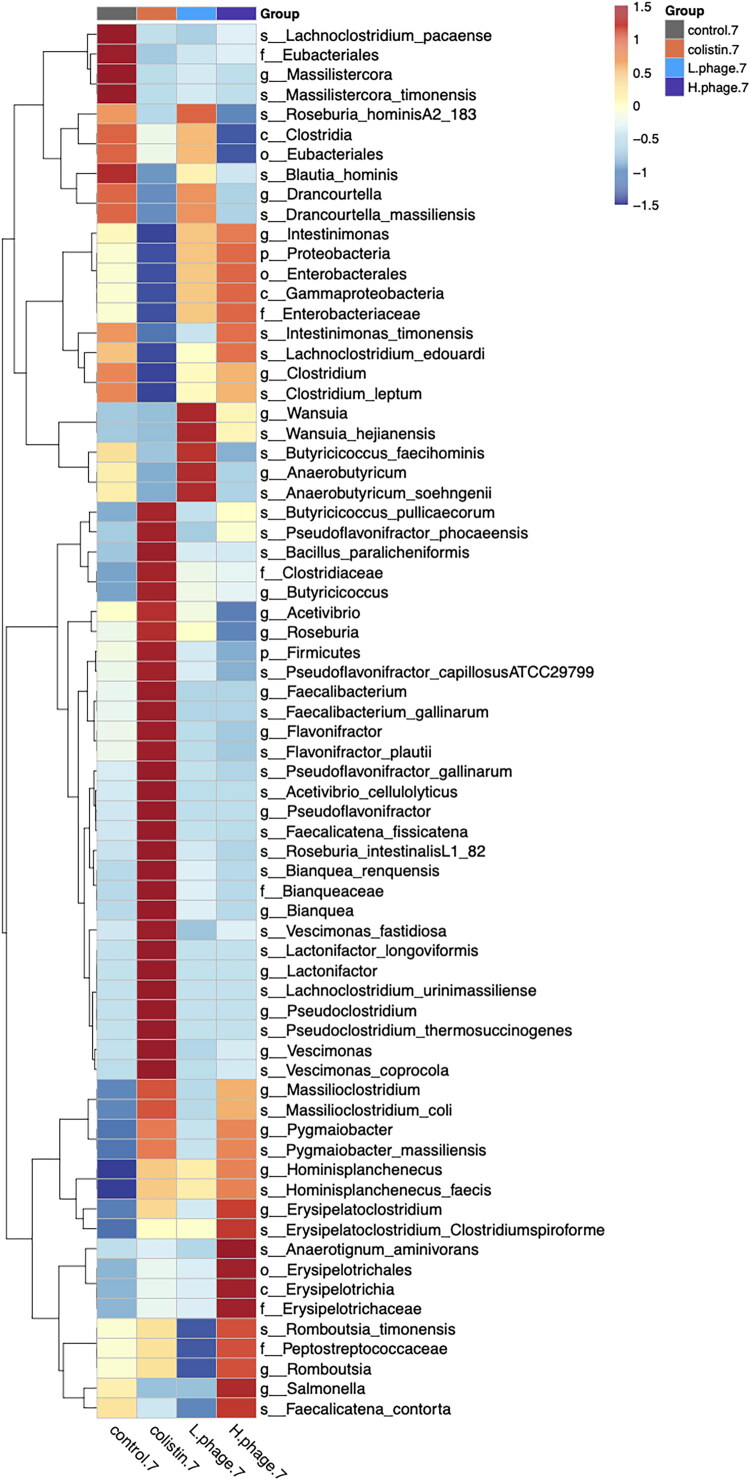
LEfSe (Linear discriminant analysis (LDA) effect size) analysis of microbiome after 7 days of treatment. Data of 1-day treatment (n = 5, H-phage n = 4) and 7-day treatment (n = 6) are used, calculated with an LDA score ≧ 3.0 were shown. All groups were *Salmonella*-challenged. Treatments: Control, autoclaved water; Colistin, 0.02% Colistin; L-phage, 1 × 10^5^ PFU/mL; H-phage, 1 × 10^8^ PFU/mL.

Figure 14 and Figure 15 present the LEfSe analysis results of treatment groups on days 1 and 7, respectively. An LDA score threshold of 3.0 was applied to identify statistically significant microbial biomarkers.

In Figure 14, the differential features are interpreted from top to bottom as follows: (1) The L-phage group showed the highest relative abundance (highlighted in red), including *Lactobacillus crispatus* (LDA = 4.70, *p* < 0.05) and *Blautia argi* (LDA = 4.23, *p* < 0.05). (2) The H-phage group uniquely showed enrichment of *Salmonella* (LDA = 3.14, *p* < 0.05) and *Paludicola psychrotolerans* (LDA = 3.16, *p* < 0.05). (3) The control group was characterized by high abundances of *Drancourtella massiliensis* (LDA = 4.14, *p* < 0.05) and *Romboutsia timonensis* (LDA = 3.54, *p* < 0.05). (4) The Colistin group showed significantly increased levels of *Faecalicatena contorta* (LDA = 3.46, *p* < 0.01) and *Roseburia intestinalis L1 82* (LDA = 3.41, *p* < 0.05), among other taxa.

In Figure 15, the microbial features are divided into four sub-clusters and described as follows: (1) The control group showed prominent enrichment of *Lachnoclostridium pacaense* (LDA = 3.43, *p* < 0.05) and *Massilistercora timonensis* (LDA = 4.51, *p* < 0.01). (2) The Colistin group showed relatively lower abundance of *Lachnoclostridium edouardi* (LDA = 3.95, *p* < 0.01) and *Clostridium leptum* (LDA = 3.09, *p* < 0.05). (3) The Colistin group also exhibited notably higher abundances of several taxa, such as *Bacillus paralicheniformis* (LDA = 3.48, *p* < 0.05), *Pseudoflavonifractor gallinarum* (LDA = 3.74, *p* < 0.05), *Faecalibacterium gallinarum* (LDA = 3.74, *p* < 0.001), *Bianquea renquensis* (LDA = 3.14, *p* < 0.001), *Lactonifactor longoviformis* (LDA = 3.18, *p* < 0.001), *Lachnoclostridium urinimassiliense* (LDA = 3.12, *p* < 0.05), and *Vescimonas coprocola* (LDA = 4.18, *p* < 0.001). (4) The H-phage group had an elevated relative abundance of *Anaerotignum aminivorans* (LDA = 3.13, *p* < 0.05), *Erysipelatoclostridium* (LDA = 3.81, *p* < 0.01), *Salmonella* (LDA = 3.09, *p* < 0.05), and *Faecalicatena contorta* (LDA = 3.38, *p* < 0.05).

## Discussion

Genomic analysis showed that vB_SalS_KY05 belongs to the genus *Tequintavirus* and carries a strictly lytic genome that lacks integrase, other temperate markers, and detectable virulence or antimicrobial resistance genes, supporting its suitability as a biocontrol candidate (Mavrich and Hatfull 2017; Philipson et al. 2018). Consistent with observations that large lytic bacteriophage genomes (> 50 kb) often encode tRNA genes (Bailly-Bechet et al. 2007), the tRNAs in vB_SalS_KY05 may help optimize codon usage or protect phage transcripts from host RNase degradation, reflecting evolutionary adaptation to diverse intracellular conditions (Van Den Berg et al. 2023). Within this genus, *Escherichia* phage T5 is a well-characterized prototype (Skutel et al. 2023; Vasquez et al. 2023), and its use of the outer membrane transporters FhuA (Heller and Braun 1982; Shin et al. 2012) suggests that vB_SalS_KY05 may exploit similar structures. Variations in O-antigen composition may further modulate adsorption efficiency (Kim et al. 2014), which is consistent with its inability to infect certain subspecies and serovars.

In bacteriophage applications, it is important to know whether phages can maintain their titer under specific conditions. Phage stability is influenced by environmental factors such as pH, temperature, and salinity, which can damage capsid or tail structures or degrade nucleic acids (Ackermann et al. 2004). Potential acid inactivation in the digestive tract has been mitigated in previous studies by administering antacids (Kuźmińska-Bajor et al. 2023) or using microencapsulation (Lorenzo-Rebenaque et al. 2022). In this study, feed buffering effects likely contributed to stability, suggesting future potential for combining such protective strategies to enhance oral delivery. Furthermore, previous research has shown that higher levels of water purification reduce total organic carbon, conductivity, and free chlorine, and that reactive free radicals in ultrapure water may destabilize viral proteins (Governal and Gerba 1997). In our study, stability was similar between reverse osmosis–treated and double-distilled water, whereas titers declined after 21 days in tap water, likely due to adsorption onto residual bacterial debris. Overall, the results indicate that vB_SalS_KY05 can be practically delivered *via* tap water at the farm level and remains stable at both room temperature and chicken body temperature.

In this research, vB_SalS_KY05 was efficiently propagated in a non-pathogenic *Escherichia coli* strain, reducing biosafety concerns associated with amplification on pathogenic hosts and facilitating large-scale production. A similar approach was described by Salim et al. (2022), who used *E. coli* ST155 to propagate *Salmonella* phages in a wastewater monitoring system; here, we extend this concept to an animal-use context and, importantly, observed that propagation in *E. coli* yielded higher phage titers than the original *Salmonella* host under our conditions. These findings suggest that choosing production hosts with both high propagation efficiency and a favorable biosafety profile is a rational way to exploit the polyvalent traits of bacteriophages.

*S.* Typhimurium typically causes mild symptoms in chickens (Barrow et al. 1987; Nair and Kollanoor Johny 2019), but flocks primarily serve as a vehicle for transmitting this zoonotic pathogen. Our results confirmed that *S.* Typhimurium can colonize the intestine and cause systemic infection; however, unexpectedly, the higher phage dose did not show improved antimicrobial ability. This differs from previous reports, where Adhikari et al. (2017) demonstrated significant *S.* Enteritidis reduction in cecum, spleen, and ovary with a 0.2% *Salmonella* phage SP-1 and STP-1 cocktail compared with 0.1% in feed, and Sarrami et al. (2023) likewise showed better effects in the 0.15% ProBe-Bac phage cocktail supplement group than in the 0.1% group. However, the dosage gap in our study was exceptionally large, so the results may not be directly comparable. In our case, the high-dose treatment differed markedly from the low-dose treatment, with *Salmonella* and reisolated phage counts showing related patterns.

The diminished efficacy observed in the H-phage group may reflect the emergence of phage-resistant bacterial subpopulations (Hsu et al. 2019). Coexistence of vB_SalS_KY05 and *Salmonella* in host tissues suggests the onset of resistance mechanisms, potentially driven by stress-induced mutagenesis (MacLean et al. 2013) or superinfection exclusion. vB_SalS_KY05 encodes a Cor lipoprotein (ORF112), homologous to mEp167’s Cor, known to block bacteriophage re-entry by masking FhuA (Arguijo-Hernández et al. 2018). Superinfection exclusion not only wastes bacteriophage particles but may also result in prophage formation, enabling long-term bacterial survival (Bucher and Czyż 2024). By contrast, a low dose of vB_SalS_KY05 effectively reduced systemic *Salmonella*, suggesting that optimal dosing supports sequential lysis and self-propagation, leading to a stable low-bacteria, low-phage equilibrium.

Noteworthy, the antibacterial effect of bacteriophages was hinted in liquid culture assays (Figure 3B), where the higher MOI treatment showed a turbidity surge in the late time point. Unlike the closed and simplified *in vitro* environment, the animal gut presents additional pressures, including competition from other microbes for ecological niches and opportunities for horizontal gene transfer that may enhance viral fitness, driving phage–bacteria coevolution (Shkoporov et al. 2022). Given that bacteriophages rely on bacteria for replication, their pharmacokinetics and pharmacodynamics are governed by density-dependent amplification and inundation thresholds rather than conventional drug kinetics, so higher input titers do not necessarily yield better bacterial clearance (Payne et al. 2000). Notably, resistance mutations induced by bacteriophage pressure often involve functional trade-offs, such as reduced growth rate, impaired biofilm formation, or increased antibiotic susceptibility (Hasan and Ahn 2022), suggesting that phage–antibiotic combinations may be a promising treatment strategy.

Chickens exhibit dynamic inflammatory and immune responses to *Salmonella* infection. Khan and Chousalkar (2020) reported that IL-1β is rapidly upregulated in the early phase, followed by pronounced fluctuations, whereas IL-6 shows a more sustained increase, underscoring its role as a multifunctional coordinator in host defense. Susceptibility also varies with genetic background, as different chicken breeds display differential sensitivity to *Salmonella* (van Hemert et al. 2006). Huang et al. (2022) observed higher serum IL-6 in the *Salmonella* phage CKT1 treated group, indicating an inflammatory response associated with phage administration. Correspondingly, low-dose vB_SalS_KY05 treatment in our study reduced pathogen load but was accompanied by sustained inflammation from day 1 to day 7, suggesting an inflammatory component attributable to phage intervention. Additionally, both the L-phage and Colistin groups showed improved albumin-to-globulin ratios on day 7 compared with day 1, indicating partial immune recovery (Abudabos et al. 2016).

Beyond antimicrobial effects, bacteriophages possess immunomodulatory potential. They may enhance macrophage phagocytosis, facilitate immune recognition *via* opsonization (Górski et al. 2012; Kaur et al. 2014), or upregulate IL-1 receptor antagonist to suppress IL-1α/β-mediated Inflammation (Van Belleghem et al. 2017). As biologically active particles containing DNA or RNA, phages can also act as pathogen-associated molecular patterns (PAMPs) recognized by innate pattern recognition receptors, triggering inflammation (Van Belleghem et al. 2018). Phage-mediated bacterial lysis may further amplify PAMPs release (Borysowski and Górski 2008). Thus, phage effects on inflammation are multifaceted and not solely dependent on bacterial clearance. Besides the direct effects of the bacteriophages, residual endotoxin was not quantified in this study. Similarly, most previous animal studies using *Salmonella* phages did not report any endotoxin removal step (Huang et al. 2022; Pelyuntha et al. 2022; Hao et al. 2023; Nicolas et al. 2023). However, endotoxin levels will need to be carefully considered before any commercial application (Abbas et al. 2022). It has been estimated that crude phage lysates can contain around 10^4^ endotoxin units (EU)/mL (1 EU ≈ 100 pg *E. coli* LPS) (Bonilla et al. 2016). Although poultry generally show higher tolerance to LPS than mammals (Reisinger et al. 2020), the potential inflammatory impact of residual endotoxin should still be taken into account.

Lastly, the microbiota composition was analyzed to investigate the impact of vB_SalS_KY05 intervention. Bacteriophages may alter microbial community structure through cascading effects within the ecosystem (Hsu et al. 2019). For example, Huang et al. (2022) used the *Salmonella* phage CKT1 and reported a beneficial shift in the gut microbiota, with higher abundances of *Lachnoclostridium*, *Lactobacillus*, and *Ruminococcus* in treated group, which was associated with more balanced body weight in chickens; but there is also a negative outcome in Hao et al. (2023), where they found higher *Fournierella* and *Escherichia shigella* level, showing a complex and inconsistent results in phage-based intervention. In our work, the SPF chickens used in this study were 1–2 weeks old, raised in a protected environment with a single feed source, resulting in a microbiota composed almost only of Firmicutes. Overall, the major top 10 genus shown barely difference after 1-day and 7-day treatments, whereas a more sensitive LEfSe analysis revealed *Lactobacillus crispatus* as a biomarker in the L-phage group after 1 day. *L. crispatus* is considered beneficial gut bacteria that can improve feed conversion, enhance immunity (Asghar et al. 2016), and modulate fat metabolism (Ding et al. 2021). Simultaneously, *Blautia coccoides* has immunomodulatory properties and promotes feed efficiency (Xie et al. 2025), suggesting that low-dose phage treatment may benefit gut microbiota composition. In contrast, *Salmonella* was identified as the biomarker for the H-phage group, followed by the control group, with lower abundance in the Colistin and L-phage groups. However, LEfSe analysis also identified *Erysipelatoclostridium* as a biomarker in the H-phage group at day 7. This genus has been reported to increase following *S.* Typhimurium infection and is linked to dysbiosis (Khan et al. 2024), raising concerns that high-dose phage treatment might not only fail to reduce *Salmonella* but also promote coexistence with harmful taxa. This work identified a beneficial effect of low-dose vB_SalS_KY05 during the early stage of infection, but no difference with control group after a week. This pattern suggests a possibility of a naturally incomplete recovery after *Salmonella* infection in chickens, and bacteriophages may exhibit different dynamics of pathogen clearance compared with antibiotics, which exert a more prolonged impact over time.

Nonetheless, the absence of a non-*Salmonella*-challenged group and a phage-only group are major limitations of this study. A non-challenged group would provide a relatively healthy baseline for evaluating the impact of the infection model, while a phage-only group would help clarify any inflammatory effects of the phage itself. In addition, the unexpected coexistence of high phage titers and *Salmonella* in the H-phage group suggests the possible emergence of phage-resistant or tolerant subpopulations, a hypothesis that warrants further investigation. In future work, we also plan to optimize phage preparation to minimize the impact of residual endotoxin, extend the observation period, and conduct trials in commercial flocks rather than SPF chicks to better reflect field conditions.

## Conclusion

This study demonstrates that the locally isolated polyvalent phage vB_SalS_KY05 exhibits potent lytic activity against *Salmonella enterica* serovars Typhimurium and Enteritidis and can be safely and efficiently produced at scale *via* host-switching to a non-pathogenic *E. coli* strain. *In vivo* trials revealed that low-dose administration effectively reduced systemic *Salmonella* infection and promoted beneficial shifts in gut microbiota composition, whereas high doses facilitated phage–bacteria coexistence and enrichment of potentially harmful taxa. Importantly, low-dose vB_SalS_KY05 intervention did not significantly alter overall cecal microbiota diversity, supporting its potential as a targeted, microbiota-sparing strategy for poultry production. These findings underscore the critical importance of dosage optimization in bacteriophage therapy and provide a practical framework for developing locally sourced bacteriophage-based solutions in poultry production.

## Supplementary Material

Supplemental Material

figure S1.png

Figure S3.tiff

Figure S2.tiff

## Data Availability

The data that support the findings of this study are available from the corresponding author upon reasonable request.
