# Supplementary material for: Bacteriophage vB_SalS_KY05 controls Salmonella in poultry without disrupting cecal microbiota composition
Source: Vet Q. 2026 Jan 21;46(1):2617464. doi: 10.1080/01652176.2026.2617464 (PMC12829423; doi:10.1080/01652176.2026.2617464)
Supplement: Supplemental Material [file TVEQ_A_2617464_SM5941.docx]

**Table S1**. Summarized the genomic features of vB_SalS_KY05.

| **vB_SalS_KY05** |  |
| --- | --- |
| GenBank accession number | PX046473 |
| Linage | *Tequintavirus* |
| Total length | 109335 bp |
| GC content | 39% |
| Coding Sequence | 171 |
| antibiotics resistant genes | - |
| virulence genes | - |

The resistance and virulence genes were analyzed from Virulence Factor Database (VFDB) and Comprehensive Antibiotic Resistance Database (CARD) via PhageScope (<https://phagescope.deepomics.org/>).

**Supplementary data**

**Figure S1.** Top 10 heatmap relative abundance on the species level.

The left part shows the outcome after 1 day of treatment (.1), and the right part is for the 7-day treatment (.7). All groups were *Salmonella*-challenged. Treatments: Control, autoclaved water; Colistin, 0.02% Colistin; L-phage, 1 × 10⁵ PFU/mL; H-phage, 1 × 10⁸ PFU/mL.

**Figure S2.** Genomic map of vB_SalS_KY05.

Data of 1-day treatment (n=5, H-phage n=4) and 7-day treatment (n=6) are used. vB_SalS_KY05 with a circular dsDNA genome for a total of 109,335 bp. The purple color indicates the infection and replication system protein, the light green represents the assembly and packaging system protein, the dark green color indicates tRNA genes, the blue indicates structural-related protein, the red color highlights lysis protein, the dark grey is for protein which has been found in bacteriophage but the function has not been fully defined yet, and the light grey stands for hypothetical protein. The direction of the arrows represents the direction of gene expression.

**Figure S3.** Functional genome map of vB_SalS_KY05.

The whole genome is separated into 3 parts: (A) 1- 30,889 bp, (B) 30,889- 78,779 bp, and (C) 78,779-109,335 bp. Colors are for functional categorization: purple for infection and replication, light green for assembly and packaging, dark green for tRNA genes, blue for structurally related protein, red for lysis, dark grey for functionally ensured protein, and light grey for hypothetical protein. Arrowhead is directed in the direction of ORF. The GC content is shown using the average of 39% as the middle.
