# Supplementary figures and images for: Bacteriophage vB_SalS_KY05 controls Salmonella in poultry without disrupting cecal microbiota composition
Source: Vet Q. 2026 Jan 21;46(1):2617464. doi: 10.1080/01652176.2026.2617464 (PMC12829423; doi:10.1080/01652176.2026.2617464)

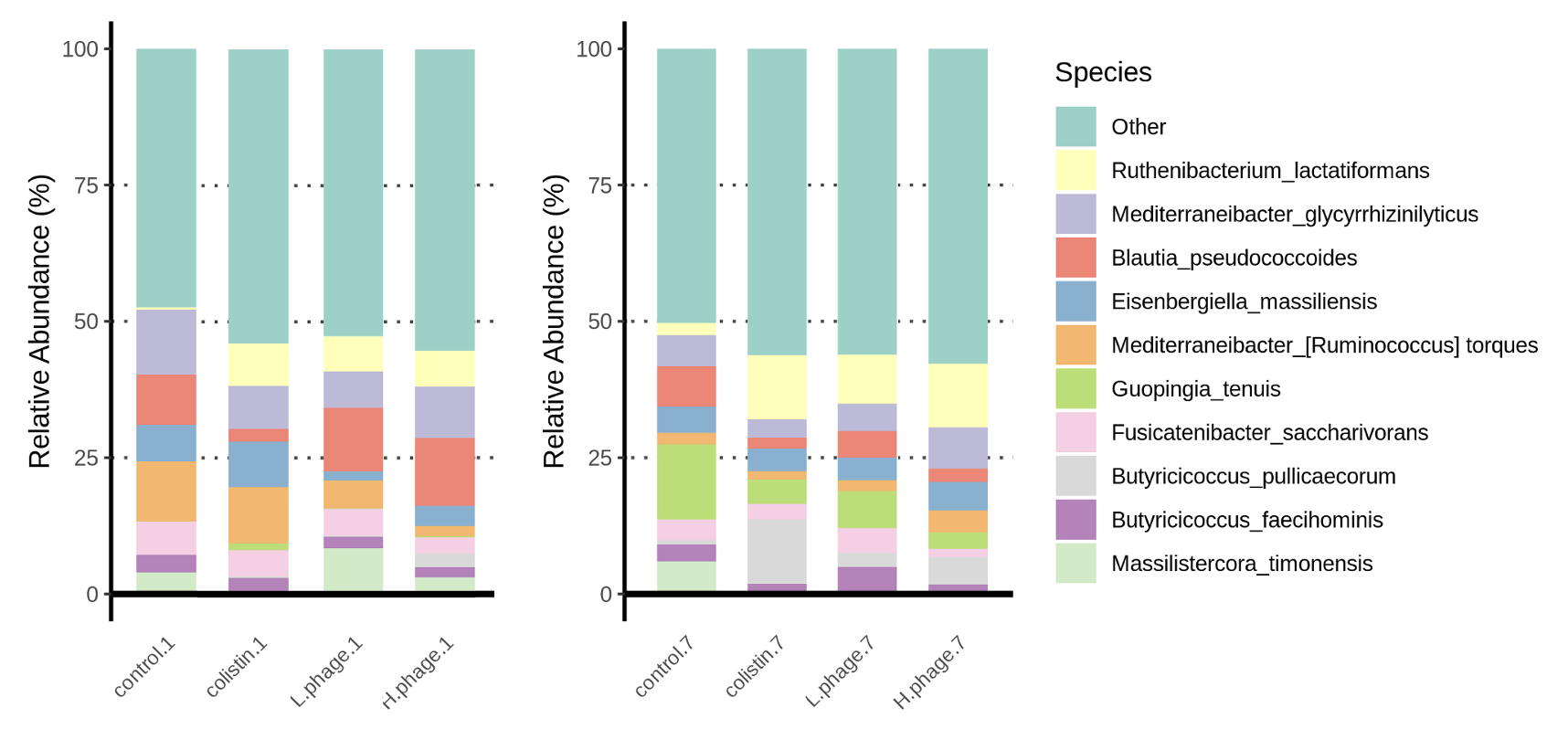

Supplement: figure S1.png [file TVEQ_A_2617464_SM7065.png]

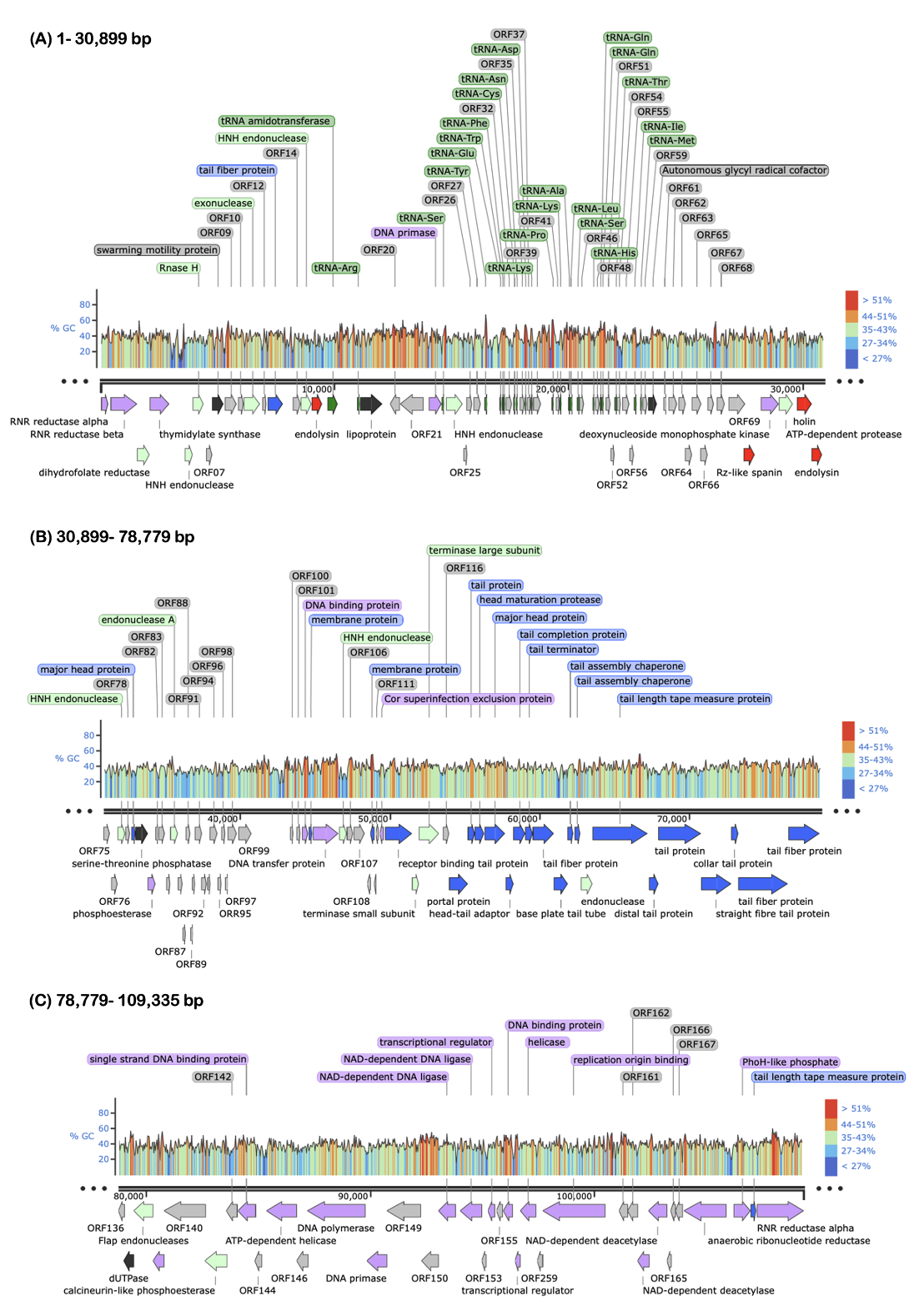

Supplement: Figure S3.tiff [file TVEQ_A_2617464_SM7064.tiff]

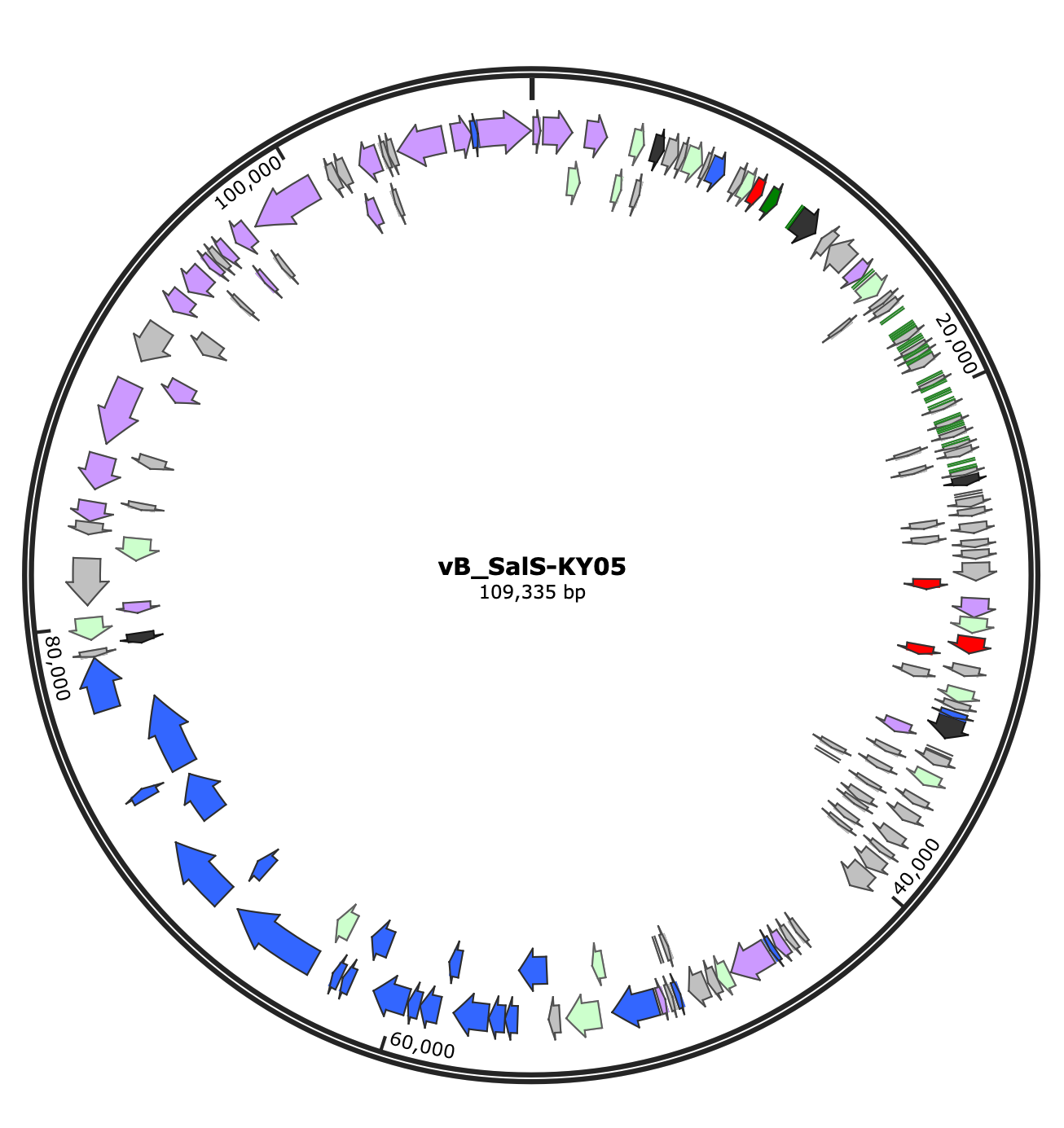

Supplement: Figure S2.tiff [file TVEQ_A_2617464_SM7062.tiff]
